# Characterisation of tumour-immune phenotypes and PD-L1 positivity in squamous bladder cancer

**DOI:** 10.1186/s12885-023-10576-0

**Published:** 2023-02-01

**Authors:** Max Jung, Michael Rose, Ruth Knuechel, Chiara Loeffler, Hannah Muti, Jakob Nikolas Kather, Nadine T. Gaisa, on behalf of the German Study Group of Bladder Cancer (DFBK e.V.)

**Affiliations:** 1 Institute of Pathology, University Hospital RWTH Aachen, Pauwelsstrasse 30, 52074 Aachen, Germany; 2 Center for Integrated Oncology Aachen Bonn Cologne Duesseldorf (CIO ABCD), Aachen, Germany; 3 Department of Medicine III, University Hospital RWTH Aachen, Aachen, Germany; 4 Else Kroener Fresenius Center for Digital Health, Medical Faculty Carl Gustav Carus, Technical University Dresden, Dresden, Germany

**Keywords:** Immune cell infiltrate, PD-L1, Squamous cell carcinoma of the bladder, Bladder cancer

## Abstract

**Aims:**

Immune checkpoint inhibitor (ICI) therapy has become a viable treatment strategy in bladder cancer. However, treatment responses vary, and improved biomarkers are needed. Crucially, the characteristics of immune cells remain understudied especially in squamous differentiated bladder cancer (sq-BLCA). Here, we quantitatively analysed the tumour-immune phenotypes of sq-BLCA and correlated them with PD-L1 expression and FGFR3 mutation status.

**Methods:**

Tissue microarrays (TMA) of *n* = 68 non-schistosomiasis associated pure squamous cell carcinoma (SCC) and *n* = 46 mixed urothelial carcinoma with squamous differentiation (MIX) were subjected to immunohistochemistry for CD3, CD4, CD8, CD56, CD68, CD79A, CD163, Ki67, perforin and chloroacetate esterase staining. Quantitative image evaluation was performed via digital image analysis.

**Results:**

Immune infiltration was generally higher in stroma than in tumour regions. B-cells (CD79A) were almost exclusively found in stromal areas (sTILs), T-lymphocytes and macrophages were also present in tumour cell areas (iTILs), while natural killer cells (CD56) were nearly missing in any area. Tumour-immune phenotype distribution differed depending on the immune cell subset, however, hot tumour-immune phenotypes (high density of immune cells in tumour areas) were frequently found for CD8 + T-cells (33%), especially perforin + lymphocytes (52.2%), and CD68 + macrophages (37.6%). Perforin + CD8 lymphocytes predicted improved overall survival in sq-BLCA while high PD-L1 expression (CPS ≥ 10) was significantly associated with higher CD3 + , CD8 + and CD163 + immune cell density and high Ki67 (density) of tumour cells. Furthermore, PD-L1 expression was positively associated with CD3 + /CD4 + , CD3 + /CD8 + and CD68 + /CD163 + hot tumour-immune phenotypes. FGFR3 mutation status was inversely associated with CD8 + , perforin + and CD79A + lymphocyte density.

**Conclusions:**

Computer-based image analysis is an efficient tool to analyse immune topographies in squamous bladder cancer. Hot tumour-immune phenotypes with strong PD-L1 expression might pose a promising subgroup for clinically successful ICI therapy in squamous bladder cancer and warrant further investigation.

**Supplementary Information:**

The online version contains supplementary material available at 10.1186/s12885-023-10576-0.

## Introduction

Pure squamous cell carcinoma (SCC) accounts for 2–5% of all bladder cancers, whereas partial squamous differentiation (MIX) is found in up to 40% of urothelial carcinomas with mixed histological features [1, 2]. Little is known, however, about the immune cell infiltrate in bladder SCC and whether different histological properties correlate with the type and quantity of the immune cells. With the approval of immune checkpoint inhibitor (ICI) treatment for bladder cancer, the effector immune cell infiltrate might play a crucial role in determining therapy success [3]. While PD-L1 testing is required for first line ICI therapy in patients who are not eligible for chemotherapy [4] and adjuvant therapy, it is not necessary in the second-line setting [5]. However, PD-L1 positivity alone does not allow a good preselection of patients who will most likely benefit from ICI therapy and drug responses may vary significantly. The open-label, international, phase 3 trial study demonstrated that treatment response of immune checkpoint inhibitor pembrolizumab was independent of the PD-L1 status [6], stressing the need for additional predictive biomarkers or a combination thereof [7, 8]. One important factor seems to be the tumour microenvironment, which has been shown to influence prognosis and ICI responsiveness in cancer of various primary cell origins [9, 10]. Cancer cells, stromal cells and immune cells engage in complex interactions which can ultimately determine cancer suppression or progression [11]. Better characterisation of the tumour microenvironment might therefore improve therapeutic stratification. Previous studies classified tumour-immune phenotypes into three different classes: ‘hot’ or ‘inflamed’ tumours (immune infiltrate in the tumour core), ‘cold’ or ‘immune desert’ tumours (no immune cell infiltration within the tumour) and ‘immune-excluded’ tumours (immune cells along tumour boundaries) [12, 13]. In urothelial bladder cancer, tumour-infiltrating lymphocytes (TILs) are associated with good prognosis [14]. High numbers of CD3 + and CD8 + tumour-infiltrating lymphocytes have been observed to predict favourable outcome in muscle-invasive urothelial carcinoma, whereas in low grade non-muscle-invasive carcinoma CD3 + and CD8 + lymphocytes were predictive of bladder cancer recurrence [15–19].

However, studies addressing the detailed immune cell infiltrates and their associations with predictive biomarkers for therapy stratification are still understudied in squamous bladder cancer. Therefore, in the present study we analysed the histology and performed manual and automatic quantification of the immune cell infiltrate of squamous bladder carcinoma. In addition, we defined distinct tumour-immune phenotypes and correlated our findings with previous data on PD-L1 expression and *FGFR3* mutation status, *PIK3CA* mutation status, ARID1A, Nectin-4 and Trop-2 expression, to investigate the potential role of immune cells as biomarkers for ICI and targeted therapy.

## Materials and methods

### Study cohort, histological evaluation and previous mutational data

Tissue microarrays (TMA) containing a total of 114 cases (*n* = 68 pure SCC, *n* = 46 MIX) from previous projects [20] were manually assessed on haematoxylin and eosin-stained slides (H&E). Tumour-infiltrating lymphocytes were evaluated according to the guidelines for TILs assessment from the International Immuno-Oncology Biomarker Working Group and semi-quantitatively categorized as none (0), few ( +), moderate (+ +) and extensive (+ + +) in intratumoural (iTILs) and stromal (sTILs) regions, respectively [21]. We used mean values for parameters of TMA slides that contained two cores per patient. Assessment of the histological parameters was done by MJ and additionally verified by an experienced pathologist (NTG). The local Ethics Committee at the RWTH Aachen Faculty of Medicine approved the retrospective, pseudonymized study of archival tissue samples and associated clinico-pathological data (RWTH EK009/12, EK455-20). Clinico-pathological characteristics of our cohort are summarised in Supplementary Table 1.

Further data on PD-L1 expression, *FGFR3* and *PIK3CA* mutation status, ARID1A, Nectin-4 and Trop-2 expression were available from previous studies of our group [20, 22–24]. PD-L1 expression had been evaluated generally for positive staining and additionally using the combined positive score (CPS), which is defined as the ratio of PD-L1–stained cells (tumour cells, lymphocytes, macrophages) to the total number of viable tumour cells multiplied by 100 [25]. We used a CPS ≥ 10 as cutoff for high PD-L1 expression consistent with the current guidelines on ICI therapy in bladder cancer [26].

### Immunohistochemical analysis

Immunohistochemistry (IHC) for T-cells (anti-CD3, anti-CD4, anti-CD8), B-cells (anti-CD79A), NK-cells (anti-CD56) and macrophages (anti-CD68, anti-CD163) was performed on TMA slides to characterise the immune cell infiltrate by subpopulations. Immune cells were scored semi-quantitatively as described above. Additionally, chloroacetate esterase staining according to routine protocols was applied to detect neutrophil granulocytes, Ki67 as a general proliferation marker and perforin to identify activated cytotoxic T-cells [27]. IHC was run on a DAKO Autostainer (DAKO, Hamburg, Germany) using DAKO EnVision^TM^FLEX system (mouse or rabbit linker and horseradish peroxidase-conjugated polymer) and DAKO Liquid DAB Substrate Chromogen according to standard protocols. Antibodies and low pH (pH 6) or high pH (pH 9) target retrieval (DAKO) are listed in Supplementary Table 2.

### Quantitative image analysis via QuPath

We used QuPath (v0.2.3), an open-source software designed for digital bioimage analysis, to analyse immune cell topographies on IHC slides [28]. The workflow consisted of TMA de-arraying, stain estimation, simple tissue detection and positive cell detection, deploying QuPath’s own integrated cell detection algorithm. Parameters for cell detection were adopted from Kather et al. [29] and manually adjusted for the different staining types in this study (see Supplementary Table 3). We then trained a Random Trees (Rtrees) Pixel Classifier by annotating representative areas of tumour and stroma and applied it to all TMAs of the study. Utilizing both positive cell detection and classification via the pixel classifier, we were able to generate the density (number of positive cells per mm^2) of positively stained cells and assign them to tumour and stromal areas. The median cell density was calculated for each marker across both tumour and stroma regions and used as a threshold for high and low density to allow classification into three distinct tumour-immune phenotypes (“hot”: high density of intratumoural TILs; “cold”: low density of intratumoural and stromal TILs; “excluded”: high density of stromal TILs but low density of intratumoural TILs) as described by Kather et al. [29]. Tumour-immune phenotypes with more than one immune cell marker were specified to analyse putative associations of markers potentially expressed by a specific cell type (like T-cells) and PD-L1 or perforin positivity.

### Statistics

Spearman-Rho test was performed to assess correlations between density and topography of different immune cells and histological and genetic characteristics. Kruskal–Wallis ANOVA, Mann–Whitney U-test and Dunn’s post hoc test were used to examine differences in immune cell densities. Pearson’s chi-square and Fisher’s exact test were deployed for analysis of immune topographies using available data of molecular and immunohistochemical markers. Furthermore, Kaplan–Meier analysis using log rank test was performed to examine the clinical impact of different immune cell subsets. Overall survival (OS) was defined as the period from surgery until death and was censored for patients without evidence of death at the last follow-up. In all our analyses, *p* values ≤ 0.05 were considered statistically significant. All statistical analyses were calculated using IBM SPSS Statistics (v27.0.0.0).

## Results

### Histopathological characteristics and immune cell infiltrate in squamous bladder cancers

**Fig. 1:**
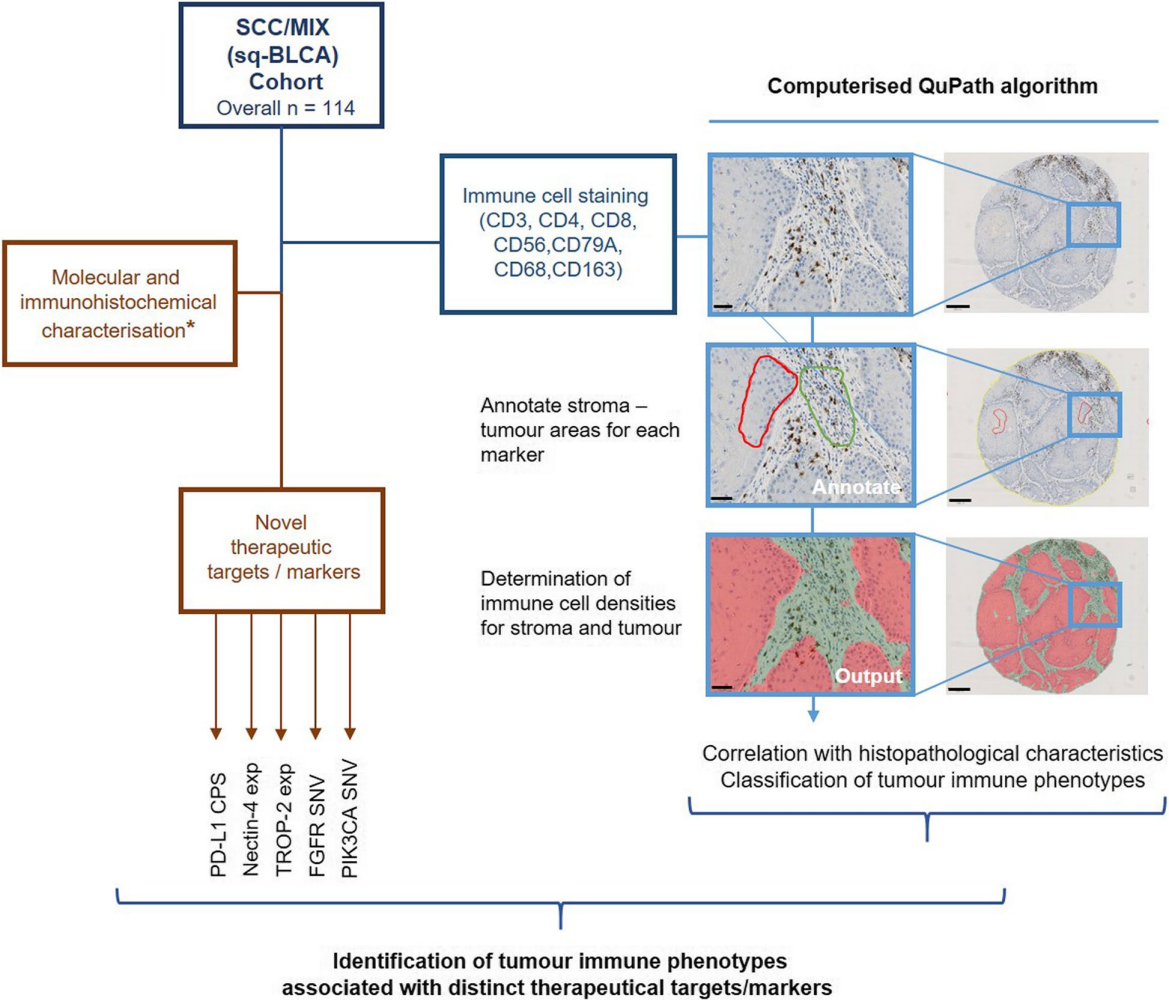
Experimental design. Workflow with multiple steps: A cohort of sq-BLCA samples (*n* = 114) already characterised for therapeutic targets in previous studies (sample numbers varies for targets; *(20, 23, 24)) was immunohistochemically stained and assessed for immune cell markers. TMA slide images were annotated for stromal and tumour areas and immune cells were automatically quantified to determine immune cell densities. Resulting immune phenotypes were correlated with therapeutic targets (or expression of marker molecules). Black scale bar (left panel): 50 µM; Black scale bar (right panel): 250 µM

The basis of our study (for experimental design see Fig. 1) was a systematic histopathological and immunohistochemical characterisation of immune cells in sq-BLCA (for cohort characteristics see Supplementary Table 1) stained by distinct markers.

**Fig. 2:**
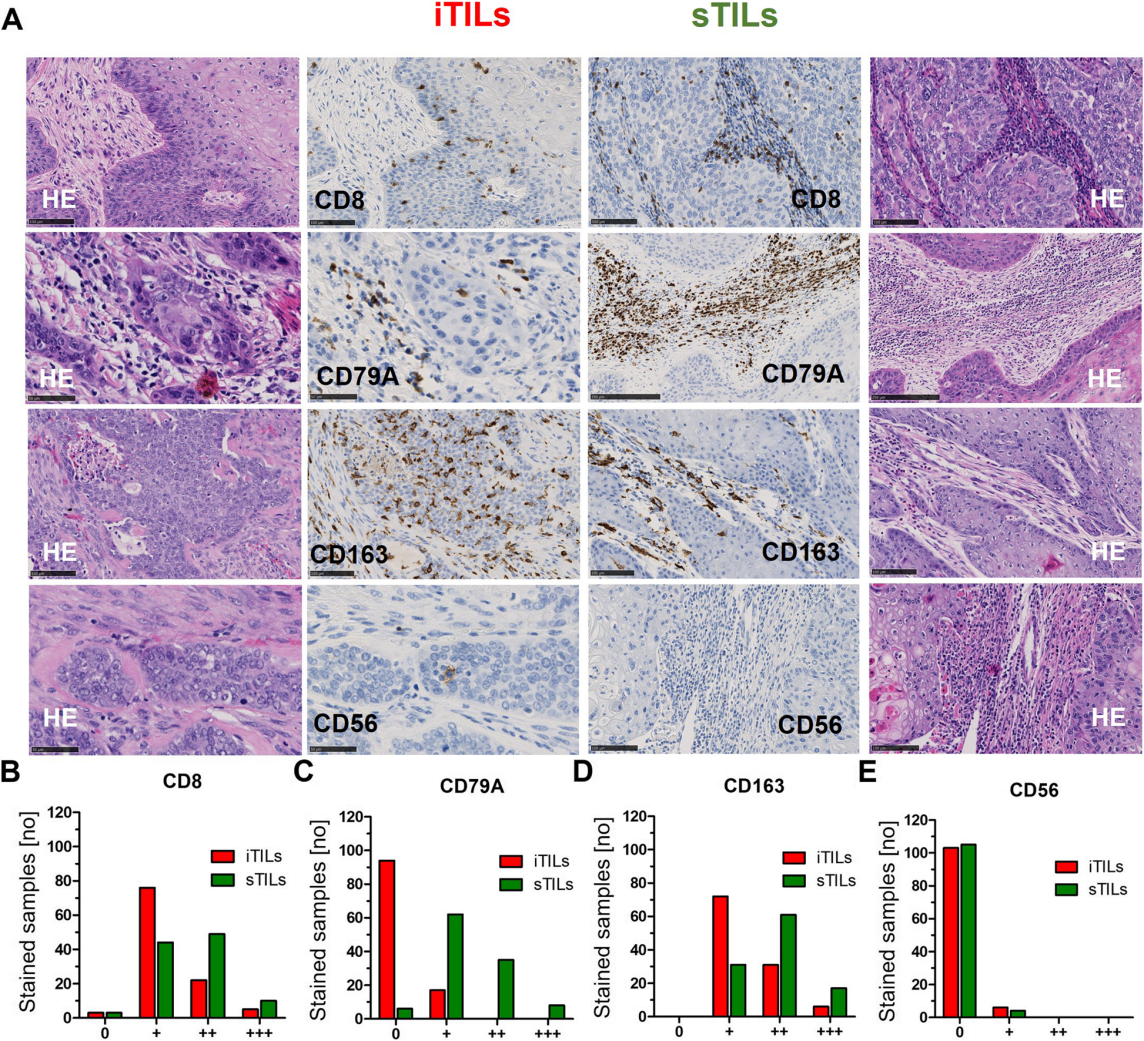
Immune cell marker expression in squamous differentiated bladder cancer (Sq-BLCA). **A** Immunohistochemical stainings of CD8, CD79A, CD163 and CD56 are shown for representative tissue cores and intratumoural (iTILs) and stromal tumour-infiltrating lymphocytes (sTILs), respectively. Tumour and stromal components are histologically represented by H&E staining. Black scale bar: 50 µM (iTILs CD79A and CD56); 100 µm (CD8, CD163, sTILs CD56); 250 µm (sTILs CD79A). (**B**-**E**) Graphs display distribution of immune cell marker expression of CD8 (**B**), CD79A (**C**), CD163 (**D**) and CD56 (**E**) in iTILs (red bars) and sTILs (green bars)

The sq-BLCA cohort was characterised by overall moderate to extensive tumour-infiltrating lymphocytes (TILs), and no significant difference was observed between pure SCC and MIX (Supplementary Fig. 1, Supplementary Table 4). Immune infiltration was generally higher in stroma (sTILs) than in tumour (iTILs) regions. B-Cells (CD79A) were found almost exclusively in stromal areas (iTILs: “0”: 94/111 (84.7%); “ + ”: 17/111 (15.3%); sTILs: “0”: 6/111 (5.4%); “ + ”: 62/111 (55.9%); “ +  + ”: 35/111 (31.5%); “ +  +  + ”: 8/111 (7.2%)) (see Fig. 2A, C). T-lymphocytes (CD3, CD4, CD8) and macrophages (CD68, CD163) were also present in tumour cell areas (iTILs) (Fig. 2A, B + D). Almost no natural killer cells (CD56) (TILs: “0”: 101/109 (92.7%); “ + ”: 8/109 (7.3%)) were observed, neither in stromal nor tumour areas (Fig. 2E).

### Computer-based algorithms allow objective and robust determination of immune cell infiltrate densities

**Fig. 3:**
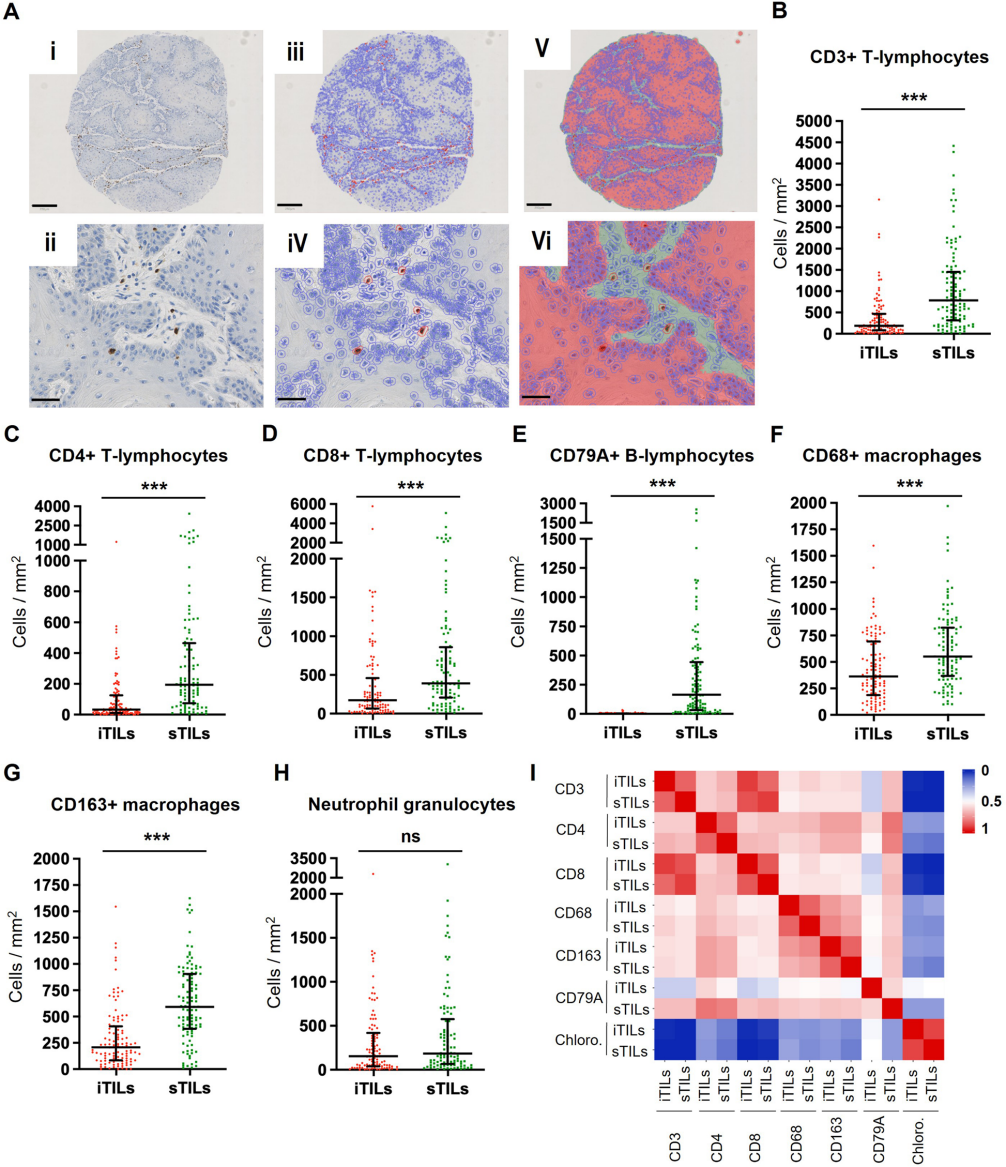
Quantification of immune cell densities in stroma and tumour areas of sq-BLCA. **A** Visualization of the computer-based analysis: (i) TMA Core stained for CD3 and (ii) detail magnification with tumour and stroma areas. (iii + iv) Positive cell detection by QuPath highlighting positively stained cells in red and non-stained cells in blue edging. (v + vi) Classifier categorizing pixels into tumour (red) and stroma (green), allowing subsequent allocation of positively stained immune cells in intratumoural and stromal TILs; Black scale bar (i, iii, v): 250 µM; Black scale bar (ii, iv, vi): 50 µM. (**B**-**H**) Immune cell density quantified by QuPath in iTILs and sTILs for CD3 + (**B**), CD4 + (**C**), CD8 + (**D**), CD79A + (**E**), CD68 + (**F**), CD163 + (**G**) and neutrophils (**H**). **I** Pairwise heatmap visualization demonstrates a strong correlation for the presence of T-cells (CD3, CD4 and CD8) as well as macrophages (CD68 and CD163); ns: not significant; ***p < 0.001

Next, we applied computer-based image analysis to automatically detect the immune cells stained by IHC (see representative images for CD3 in Fig. 3A) and to specify cell densities (Fig. 3B-H), allowing more precise and objective quantification of the immune cell density than the highly variable semiquantitative scoring by histopathology trained and non-trained individuals (see Supplementary Fig. 2). Since CD56 was rarely stained in sq-BLCA, NKT-cells were left out for further analysis with QuPath. A significantly increased immune cell density was verified for all analysed markers in sTILs compared to iTILs (e.g., CD8: iTILs median density: 172.4 cells/mm^2^ vs. sTILs density: 391.9 cells/mm^2^) except for chloroacetate esterase positive granulocytes (Fig. 3H) and perforin positive lymphocytes (Supplementary Fig. 3). We further confirmed a highly decreased intratumoural CD79A B-cell density (iTILs median density: 1.2 cells/mm^2^ vs. sTILs density: 164.0 cells/mm^2^).

Non-parametric Spearman-rank test demonstrated a strong correlation for T-cell markers (Fig. 3I), especially for CD3 and CD8 in both iTILs and sTILs (iTILs r: 0.900, *p* < 0.001; sTILs: r: 0.913, *p* < 0.001;). A close correlation was also observed between macrophage markers CD68 and CD163 (iTILs r: 0.655, *p* < 0.001; sTILs: r: 0.633, *p* < 0.001;). Chloroacetate esterase staining reflecting neutrophil granulocytes did not significantly correlate with any of the other immune cell markers.

We subsequently evaluated associations of clinico-pathological characteristics and immune cell densities. Increased density of CD8 + lymphocytes was significantly associated with mixed squamous differentiated bladder cancers (*p* = 0.028) (Table 1). This relationship was matched by perforin + lymphocytes and MIX bladder cancer (*p* = 0.006) (Supplementary Table 5). As expected, poor grading (G3) was associated with high Ki67 expression (*p* = 0.016), whereas – in an unthought manner—lymphatic invasion was associated with low Ki67 expression (*p* = 0.019) (Supplementary Table 6). Low CD79A + B-cell lymphocyte infiltration was associated with older age (*p* = 0.034). No other significant associations were observed (Supplementary Tables 5–12).

**Table 1:** Clinico-pathological parameters associated with CD8 density

	**CD8 density**^b^			
	***n***^*a*^	**low**	**high**	***P*-value**^c^
***Parameter:***				
Age at diagnosis				
< 68 years	50	23	27	0.616
≥ 68 years	53	27	26	
Gender				
male	48	24	24	0.852
female	54	26	28	
Tumour subtype				
pure SCC	65	38	27	**0.028**
mix SCC	41	15	26	
Histological tumour grade				
G1-G2	30	16	14	0.447
G3-G4	71	32	39	
Tumour stage				
pT1-pT2	15	5	10	0.288
pT3-pT4	83	40	43	
Nodal status				
pN0	62	29	33	0.597
pN +	20	8	12	
Lymphatic invasion				
L0	38	18	20	0.707
L1	19	8	11	

^a^Only patients with primary sq-BLCA were included; ^b^density in cells / mm^2^; median value of the overall sample (291.6 / mm^2^) as cut-off for low and high density; ^c^Pearson's chi-square test; Significant *P*-values are marked in bold face. Please note: sample numbers may vary between analyses due to limitations of usable TMA cores depending on the staining

### Classification of tumour-immune phenotypes reveals ‘Immune excluded’ tumours to be the most frequent in SCC

**Fig. 4:**
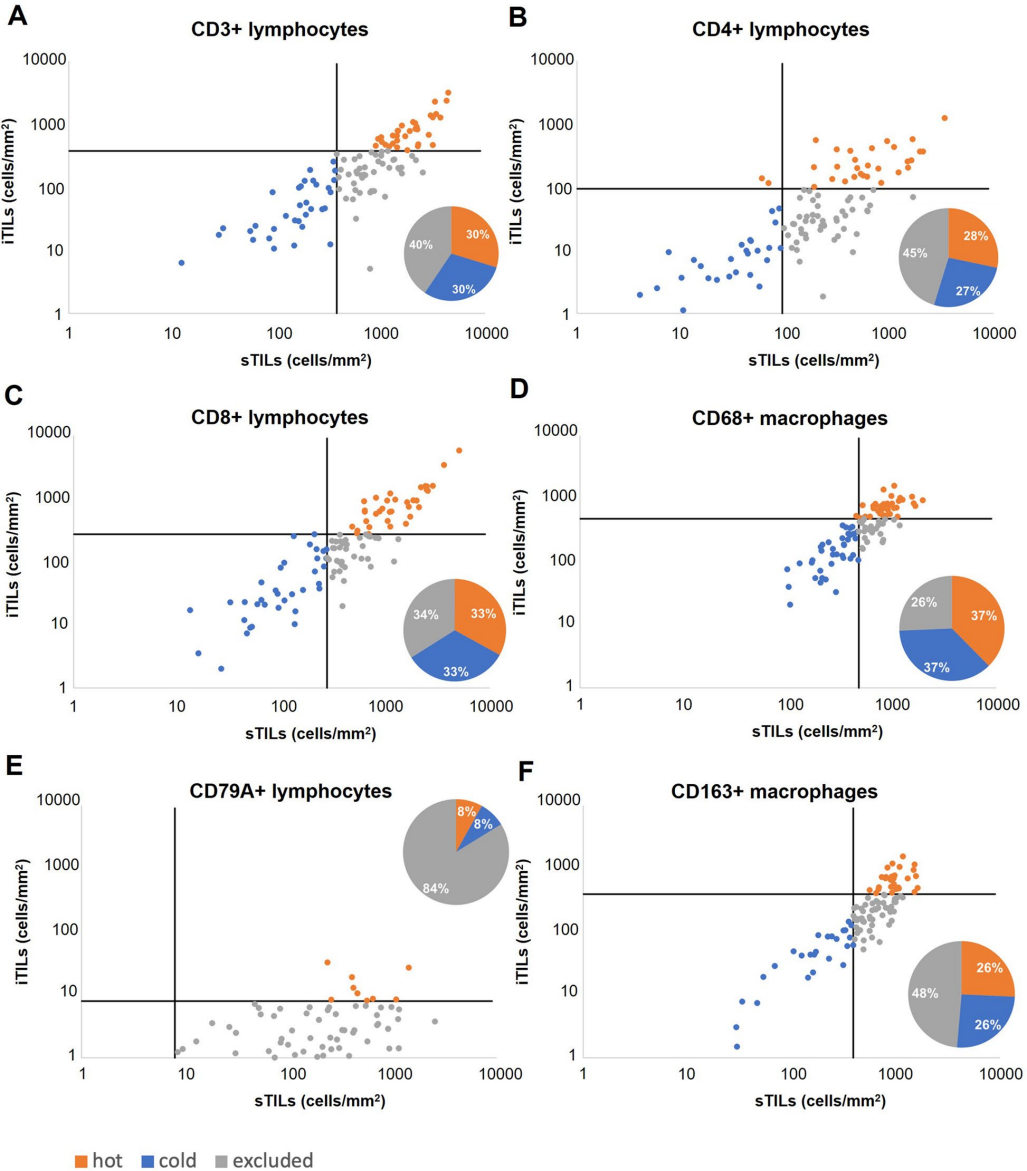
Tumour-immune topography: Scatter plots of tumour and stromal cell densities of (**A**) CD3, (**B**) CD4, (**C**) CD8, (**D**) CD68, (**E**) CD79A and (**F**) CD163 immune cells and tumour-immune phenotype distributions are illustrated. Classification of tumour-immune phenotypes are based on median cell densities across tumour and stroma regions for each marker

Characterising specific immune phenotypes based on quantified densities, we found heterogeneous distributions of immune topographies for different immune cells (Fig. 4). For CD3 + and CD4 + T-cells ‘excluded’ was the most frequent phenotype. Almost half of the tumours were CD4-excluded (45.3%) and the remaining split into hot (28.3%) and cold (26.4%) tumours. CD3 + showed less excluded tumours (40.5%) but a similar distribution for hot (29.7%) and cold (29.7%) tumours.

For CD8 + T-cells, there was a nearly even distribution of tumour-immune phenotypes (33.0% CD8-hot; 33.0% CD8-cold; 34.0% CD8-excluded). Perforin + activated cytotoxic lymphocytes displayed the highest proportion of ‘hot’ immune phenotypes (52.2%). Since B-cells were predominantly found in stromal regions, the vast majority of tumours were CD79A-excluded (84.5%). Tumour-associated macrophages showed differences in their immune phenotype distribution with regards to the analysed cell types. While CD68-hot (37.6%) and CD68-cold (36.7%) were more common than CD68-excluded (25.7%), almost half (48.6%) were CD163-excluded. There was no significant difference in tumour-immune phenotype distribution between mixed and pure carcinomas except for CD163 (*p* = 0.007), where mixed carcinoma had a higher proportion of CD163-excluded tumours whereas pure SCC showed a higher proportion of CD163-cold tumours.

### Immune cell phenotypes with infiltrating T-cell lymphocytes and macrophages were closely associated with strong PD-L1 expression

Since we aimed to analyse different immune cell subsets and distinct tumour-immune phenotypes for therapeutic stratification in sq-BLCA, we examined close associations with molecular and immunohistochemical markers.

An inverse relationship was observed for activating *FGFR3* mutations and CD8 + T-cells (*p* = 0.039), especially perforin + lymphocytes (*p* = 0.022) and CD79A + B-lymphocytes (*p* = 0.036) (Table 2). Interestingly, PD-L1 expression was significantly associated with CD3 + (*p* = 0.019) and CD8 + (*p* = 0.020) T-cell as well as CD163 + (*p* = 0.046) macrophage density (Table 3) and Ki67 density (*p* = 0.049) (Supplementary Table 13). Focusing on distinct ‘hot’, ‘cold’ and ‘excluded’ tumour-immune phenotypes with combined immune cell markers that are known to be specific for distinct cell types, we confirmed a close association of PD-L1 positive sq-BLCA (CPS ≥ 10) with CD3 + /CD4 + (*p* = 0.022) and CD3 + /CD8 + T-cell (*p* = 0.021) as well as CD68 + /CD163 + macrophage (*p* < 0.001) hot immune phenotypes (Table 4). When considering PD-L1 expression (TPS: 0% vs > 0%) on tumour cells only, we could still confirm significant associations for most immune cells, while T-cells barely missed significance in this setting (Supplementary Table 14).

**Table 2:** FGFR3 mutation associated with immune cell densities

	**FGFR3 mutation**			
	***n***^*a*^	**neg**	**pos**	***P*-value**^b^
***Immune cell markers:***				
CD3				
≤ 582.3/mm^2^	26	22	4	0.387
> 582.3/mm^2^	36	33	3	
CD4				
≤ 141.5/mm^2^	29	26	3	0.964
> 141.5/mm^2^	28	25	3	
CD8				
≤ 291.6/mm^2^	25	20	5	**0.039**
> 291.6/mm^2^	32	31	1	
CD68				
≤ 493.0/mm^2^	28	27	1	0.121
> 493.0/mm^2^	32	27	5	
CD79A				
≤ 85.9/mm^2^	30	24	6	**0.036**
> 85.9/mm^2^	32	31	1	
CD163				
≤ 462.1/mm^2^	32	27	5	0.307
> 462.1mm^2^	28	26	2	
Perforin				
≤ 9.0/mm^2^	17	13	4	**0.022**
> 9.0/mm^2^	33	32	1	

^a^Only patients with primary sq-BLCA were included; ^b^Pearson’s chi-square test; Significant *P*-values are marked in bold face. Please note: sample numbers may vary between analyses due to limitations of usable TMA cores depending on the staining

**Table 3:** PD-L1 22C3 CPS associated with immune cell densities

	**PD-L1 22C3 CPS**^**b**^			
	***n***^*a*^	** < 10**	** ≥ 10**	***P*-value**^c^
***Immune cell markers:***				
CD3				
≤ 582.3/mm^2^	49	46	3	**0.019**
> 582.3/mm^2^	48	37	11	
CD4				
≤ 141.5/mm^2^	48	44	4	0.061
> 141.5/mm^2^	45	35	10	
CD8				
≤ 291.6/mm^2^	46	43	3	**0.020**
> 291.6/mm^2^	46	35	11	
CD68				
≤ 493.0/mm^2^	50	46	4	0.051
> 493.0/mm^2^	45	35	10	
CD79A				
≤ 85.9/mm^2^	50	45	5	0.200
> 85.9/mm^2^	47	38	9	
CD163				
≤ 462.1/mm^2^	51	47	4	**0.046**
> 462.1/mm^2^	45	35	10	
Perforin				
≤ 9.0/mm^2^	43	39	4	0.271
> 9.0/mm^2^	40	33	7	

^a^Only patients with primary sq-BLCA were included; ^b^Combined positive score (CPS) according to Kulangara et al. (25); ^c^Pearson’s chi-square test; Significant *P*-values are marked in bold face. Please note: sample numbers may vary between analyses due to limitations of usable TMA cores depending on the staining

**Table 4:** PD-L1 22C3 CPS associated with tumour-immune phenotypes

	**PD-L1 22C3 CPS**^**b**^			
	***n***^*a*^	** < 10**	** ≥ 10**	***P*-value**^c^
***Immune cell markers:***				
Immune topography T-cells (CD3/CD4)				
hot	28	19	9	**0.022**
cold	27	25	2	
excluded	38	35	3	
Immune topography T-cells (CD3/CD8)				
hot	29	20	9	**0.021**
cold	29	26	3	
excluded	34	32	2	
Immune topography B-cells (CD79A)				
hot	7	4	3	
cold	9	8	1	0.099
excluded	81	71	10	
Immune topography macrophages (CD68/CD163)				
hot	28	18	10	** < 0.001**
cold	30	26	4	
excluded	37	37	0	
Immune topography all immune cells				
hot	28	18	10	**0.002**
cold	31	28	3	
excluded	32	31	1	

^a^Only patients with primary sq-BLCA were included; ^b^Combined positive score (CPS) according to Kulangara et al. (23); ^c^Fisher’s exact test; Significant *P*-values are marked in bold face. Please note: sample numbers may vary between analyses due to limitations of usable TMA cores depending on the staining

### Activated perforin + cytotoxic T-cells predict survival with CD8 + density

**Fig. 5:**
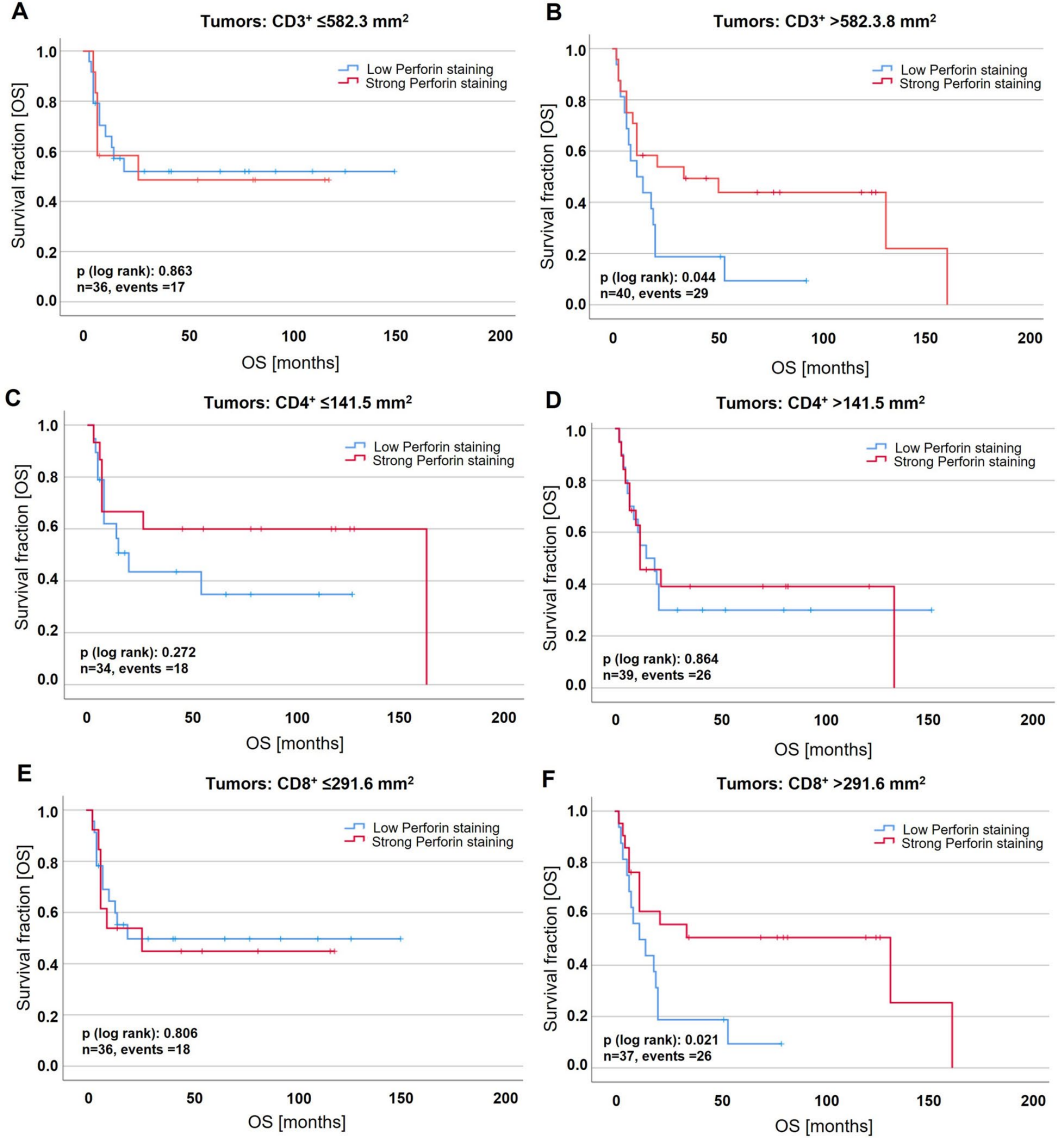
Perforin predicts overall survival (OS) in squamous bladder cancer depending on the extent of cytotoxic T-cell infiltrate. Univariate Kaplan–Meier survival curves are shown for perforin in tumours with low/high densities of T-cell markers CD3 (**A-B**), CD4 (**C-D**) and CD8 (**E**–**F**). Median cell densities of the overall sample were used as a threshold for low and high densities for each marker (CD3: 582.3 per mm^2^; CD4: 141.5 per mm^2^; CD8: 291.6 per mm^2^; Perforin: 9.0 per mm^2^). Vertical lines: censored cases

To detect immune cells with active cytotoxic functions, T-cells were further stratified by perforin staining (Supplementary Fig. 3 and Table 5). Univariate Kaplan–Meier analyses revealed significantly better overall survival for patients showing high density of perforin + lymphocytes with high densities of CD3 + (mean OS: 73.8 months ± 15.4; *p* = 0.044) and CD8 + T-cells (mean OS: 81.6 months ± 17.0; *p* = 0.021) compared to low perforin density with high densities of CD3 + (mean OS: 23.2 months ± 6.9) and CD8 + (mean OS: 21.9 months ± 6.0) T-cells (Fig. 5 A-B, 5E-F). Perforin + lymphocytes had no predictive impact across stratification of the other immune cell markers (see representatively for CD4 in Fig. 5C-D).

**Table 5:** Ki67 and perforin density associated with tumour-immune phenotypes

	**Ki67 density**^**b**^				**Perforin density**^**b**^			
	***n***^*a*^	**low**	**high**	***P*-value**^c^	***n***^**a**^	**low**	**high**	***P*-value**^**c**^
***Immune cell markers:***								
Immune topography T-cells (CD3/CD4)								
hot	26	8	18	0.102	26	9	17	**0.049**
cold	29	17	12		28	19	9	
excluded	37	19	18		35	17	18	
Immune topography T-cells (CD3/CD8)								
hot	27	7	20	**0.025**	26	7	19	**0.006**
cold	31	18	13		30	21	9	
excluded	34	19	15		33	17	16	
Immune topography B-cells (CD79A)								
hot	9	3	6	0.317	8	5	3	0.504
cold	7	5	2		6	4	2	
excluded	80	39	41		78	37	41	
Immune topography macrophages (CD68/CD163)								
hot	27	11	16	0.224	27	12	15	0.165
cold	29	18	11		28	18	10	
excluded	38	17	21		36	15	21	
Immune topography all immune cells								
hot	28	8	20	**0.039**	27	11	16	0.292
cold	32	16	16		31	19	12	
excluded	31	19	12		30	15	15	

^a^Only patients with primary sq-BLCA were included; ^b^density in cells / mm^2^; median value of the overall sample (Ki67: 509.5/mm^2^; Perforin: 9.0/mm^2^) as cut-off for low and high density; ^c^Pearson's chi-square test; Significant *P*-values are marked in bold face. Please note: sample numbers may vary between analyses due to limitations of usable TMA cores depending on the staining

There was no significant relationship of Nectin-4 or Trop-2 expression, *PIK3CA* mutations and immune cell density for any marker (Supplementary Table 15).

## Discussion

Squamous bladder cancer may develop either from urothelial carcinoma as squamous re-/trans-differentiation or from neoplastic transformation out of squamous metaplasia [30, 31]. The molecular mechanisms leading to progression of squamous metaplasia into squamous carcinoma have yet to be fully revealed, but chronic inflammation may play an important role in the carcinogenesis of SCC [32]. Inflammation has also been observed to contribute to tumour development in urothelial carcinoma even though the association has been proposed stronger for squamous cell than for urothelial carcinoma [33, 34]. Although part of the inflammation response, a comprehensive evaluation of immune infiltration profiles known to promote an immune-inflamed tumour phenotype has been missing for SCC so far [12]. Eismann et al. recently analysed immune cell infiltration of T-cells (CD3, CD4, CD8) and B-cells (CD20) and their prognostic impact in squamous bladder cancer, demonstrating improved overall survival in tumours with high CD3 + , CD4 + , CD8 + and CD20 + infiltration [35]. In 2017, Robertson and colleagues have already given first but rough insight into immune infiltration of squamous bladder cancers presenting clear lymphocyte infiltration (with or without co-infiltration of eosinophils and neutrophils) in more than 45% of sq-BLCA (overall *n* = 42) [36]. Additional 26% of sq-BLCA were characterised by at least minimal lymphocyte infiltration, while *n* = 12 (29%) lacked any immune infiltration. However, detailed description has not been given and data on spatial distribution of immune cells, evaluation of macrophages, analysis of tumour-immune phenotypes and associations with molecular and immunohistochemical markers are missing. In contrast, we aimed to define immune topographies and distinct tumour-immune phenotypes in association with molecular markers in sq-BLCA using a machine-learning approach as basis for therapy stratification.

Tumour immune cell infiltrates in general are characterised by heterogeneous immune cell types, as described in many tumour entities such as breast cancer [37, 38], melanoma [39], lung cancer [40, 41], colorectal cancer [42] but also squamous tumour entities like head and neck carcinoma [43–45] and oesophageal squamous cell carcinoma [46]. However, some immune cells may be more abundant than others, and T-lymphocytes are generally the main component of the tumour microenvironment [47]. In line with these studies, we observed a heterogeneous immune cell infiltrate consisting of mostly stromal T- and B-lymphocytes as well as macrophages.

Focusing on biomarkers and targets to predict novel therapeutic strategies, we observed a negative correlation between *FGFR3* mutation status and CD8 + , perforin + and CD79A + cell density. Fibroblast growth factor receptors (FGFRs) are involved in regulating cell proliferation, differentiation and migration [48] and FGFR inhibitors evolved as first targeted therapy applying erdafitinib in advanced urothelial bladder cancer [49]. *FGFR3* mutation has previously been linked to gene signatures of T-cell immune exclusion in urothelial bladder cancer [50] and our observed negative correlation points towards the same direction for sq-BLCA. Interestingly, a phase 2 study of erdafitinib identified a subset of patients treated with ICI who ultimately had higher response rates upon erdafitinib treatment, supporting a link between immune cell infiltration and FGFR3 (signalling) which has not been understood so far [51]. In another recently published study, inhibition of FGFR3 has been shown to increase PD-L1 expression, which then leads to suppression of T-cell-mediated immune responses [52]. However, *FGFR3* mutation does not influence response to ICI therapy in metastatic urothelial cancer [53]. In turn, PD-L1 expression is thought to predict response to ICI therapy in various tumour entities including squamous differentiated bladder cancer [20, 54].

Additionally, we further focused on PD-L1 as a predictive marker for ICI therapy and its association with immune cell infiltration. PD-L1 has been shown to be expressed by tumour cells in addition to tumour-associated antigen-presenting cells such as macrophages and T-cells [55, 56]. Robertson et al. showed increased *CD274* (PD-L1) mRNA expression in distinct molecular subtypes of urothelial bladder cancer, especially in the Ba/Sq subtype, which had a substantial fraction (42%) of tumours with squamous histology [36, 57]. Immune checkpoint inhibitor therapy has therefore been proposed as an appropriate therapeutic option in basal and squamous bladder cancer.

Nevertheless, PD-L1 expression as an exclusive predictive biomarker has its limitations, with some patients showing deviating response to anti-PD-L1/PD-1 therapy when predicted by PD-L1 expression levels [7, 58, 59]. While Balar et al. demonstrated highest ORR in patients with strong PD-L1 (> 10%) expression when treated with pembrolizumab, there are various studies showing ICI efficacy independently of PD-L1 status [6, 60, 61]. Thus, additional factors must play a role and the tumour-immune infiltration might be of clinical significance [62]. In our study, we identified distinct tumour-immune phenotypes and revealed different immune topographies for the analysed immune cells, indicating differences in terms of quality and quantity of immune responses. Tumour-immune phenotypes may also vary significantly between different tumour types. Urothelial bladder cancer has previously been shown to comprise mostly CD3 + excluded tumours but a nearly identical distribution of CD8 + phenotypes, whereas tumour entities such as melanoma, lung cancer and head and neck squamous carcinoma displayed higher proportions of CD3 + hot and CD8 + hot tumours [29]. In urothelial cancer, increased levels of PD-L1 expression have previously been associated with immune “hot” tumours, which could therefore pose an immune phenotype especially suited for immunotherapy [63].

Both CD3 + /CD4 + and CD3 + /CD8 + T-cell as well as CD68 + /CD163 + hot tumours were significantly associated with high PD-L1 expression scores, which might be relevant for therapy of squamous bladder cancer with pembrolizumab. In line with Eismann et al. [35], we could show improved overall survival in tumours with high perforin + lymphocyte density when also infiltrated with high densities of CD3 + and CD8 + lymphocytes, respectively, which might indicate a particularly strong and active immune response. This is in line with recent findings in head and neck squamous cell carcinoma, where high perforin expression also predicted improved OS and was correlated with extensive immune infiltration [64]. Erlmeier et al. recently demonstrated immune phenotypes of distant metastases of urothelial carcinoma predicting durable ICI responses [62]. In general, immune-inflamed tumours have been shown to elicit better response to ICI therapy [65–67]. It is hypothesized that in these immune cell infiltrated phenotypes tumour cells suppress an already existing anti-tumour immune response, which may then be reactivated by anti-PD-L1/PD-1 therapy [12]. In contrast, immune-excluded phenotypes, where abundant immune cells retain in the stroma, clinical responses of ICI treatment are uncommon since the immune cells do not infiltrate the tumour [12]. Hanna and colleagues have shown that CD8 + cell infiltrates predicted anti-PD-1/L1 benefit in head and neck cancer [68]. Early studies by Lyford-Pike et al. postulated an adaptive immune resistance created by the PD-1:PD-L1 interaction between CD8 + tumour-infiltrating lymphocytes and associated cancer cells in HNSCC [69]. Thus, profiles involving a co-occurrence of both infiltrating CD8 + T-cells and PD-L1 expressing tumour cells as shown in our study may indicate subgroups which could potentially benefit from ICI therapy.

## Conclusions

In our study, we used computer-based image analysis as an efficient tool to analyse immune topographies in squamous bladder cancer. We suggest that PD-L1 positive CD3 + /CD4 + , CD3 + /CD8 + and CD68 + /CD163 + hot tumour-immune phenotypes may present promising subgroups for immune checkpoint therapy in squamous bladder cancer. The immune cell infiltrate warrants further investigation as a predictive biomarker of response to ICIs.

### Limitations

However, due to the rare prevalence of (pure) squamous bladder cancer our cohort number is limited while considering serial slides and TMA cores for immunohistochemical staining do not reflect the heterogeneity of analysed tumours. Therefore, after analysing both tumour groups separately we also pooled pure squamous cell carcinomas and mixed urothelial carcinomas with substantial squamous differentiation for enhanced statistical power. This of course does not reflect possible differences in clinical behaviour, but since we used only mixed carcinomas with substantial squamous differentiation (> 70% squamous tumour parts, lots of patients with urothelial CIS (which are pure squamous cell carcinomas according to WHO 2022)) this might be relative. All analyses are of correlative nature and can only reveal association, but not causation. Further, our analyses are of retrospective nature only and suffer from limited clinical annotations and missing therapeutic data*.*

### Outlook

In order to validate the impact of tumour immune phenotypes and the overall immune cell infiltrate, prospective clinical trials with comprehensive biomarker analysis beyond PD-L1 expression and therapeutic data of larger and multicentre cohorts of pure squamous cell carcinomas and mixed urothelial carcinomas with squamous differentiation are needed. Results should also be correlated to findings in pure urothelial carcinoma patients without any squamous differentiation. Only after positive findings are confirmed might computer-based evaluation of immune phenotypes or at least semi-quantitative scoring of the immune cell infiltrate in addition to PD-L1 expression become an important component of histopathology reports.

## Supplementary Information

**Additional file 1:** **Supplementary Figure 1.** Semi-quantitative scoring of tumour-infiltrating lymphocytes and differentiation into intratumoural TILs and stromal TILs based on H&E slides.

**Additional file 2:** **Supplementary Figure 2.** Quantification of stained immune cells in stromal and tumour areas of three tumour cores by *n*=9 independent individuals.

**Additional file 3:** **Supplementary Figure 3.** Immunohistochemical staining of Ki67 and Perforin. Positively stained cell density quantified by QuPath in tumour and stroma for Ki67 and iTILs and sTILs for Perforin.

**Additional file 4:** **Supplementary Table 1.** Study characteristics of the tissue microarray cohort. **Supplementary Table 2.** Antibodies used for immunohistochemistry. **Supplementary Table 3.** QuPath cell detection parameters. **Supplementary Table 4.** Semi-quantitative scoring of TILs in pure and mix SCC. **Supplementary Table 5.** Clinico-pathological parameters associated with Perforin density. **Supplementary Table 6.** Clinico-pathological parameters associated with Ki67 density. **Supplementary Table 7.** Clinico-pathological parameters associated with CD3 density. **Supplementary Table 8.** Clinico-pathological parameters associated with CD4 density. **Supplementary Table 9.** Clinico-pathological parameters associated with CD68 density. **Supplementary Table 10.** Clinico-pathological parameters associated with CD79A density. **Supplementary Table 11.** Clinico-pathological parameters associated with CD163 density. **Supplementary Table 12.** Clinico-pathological parameters associated with neutrophil granulocytes density. **Supplementary Table 13.** PD-L1 22C3 CPS and FGFR3 mutation status associated with Ki67 density. **Supplementary Table 14.** PD-L1 22C3 tumour cells expression associated with tumour-immune phenotypes. **Supplementary Table 15.** Nectin-4, Trop-2 expression and PIK3CA mutation status association with immune cell densities.

## Data Availability

The datasets used and/or analysed during this study are available from the corresponding author upon reasonable request.

## Abbreviations

CPS: Combined positive score
FGFR: Fibroblast growth factor receptor
H&E: Hematoxylin and eosin
ICI: Immune checkpoint inhibitor
IHC: Immunohistochemistry
PD-L1: Programmed Cell Death 1 Ligand 1
sq-BLCA: Squamous bladder cancer
SCC: Squamous cell carcinoma
TILs: Tumour-infiltrating lymphocytes
sTILs: Stromal TILs
iTILs: Intratumoural Tils
TMA: Tissue microarray
