# Supplementary material for: Characterisation of tumour-immune phenotypes and PD-L1 positivity in squamous bladder cancer
Source: BMC Cancer. 2023 Feb 1;23:113. doi: 10.1186/s12885-023-10576-0 (PMC9890720; doi:10.1186/s12885-023-10576-0)
Supplement: Supplementary file 1 — Additional file 1: Supplementary Figure 1. Semi-quantitative scoring of tumour-infiltrating lymphocytes and differentiation into intratumoural TILs and stromal TILs based on H&E slides. [file 12885_2023_10576_MOESM1_ESM.docx]

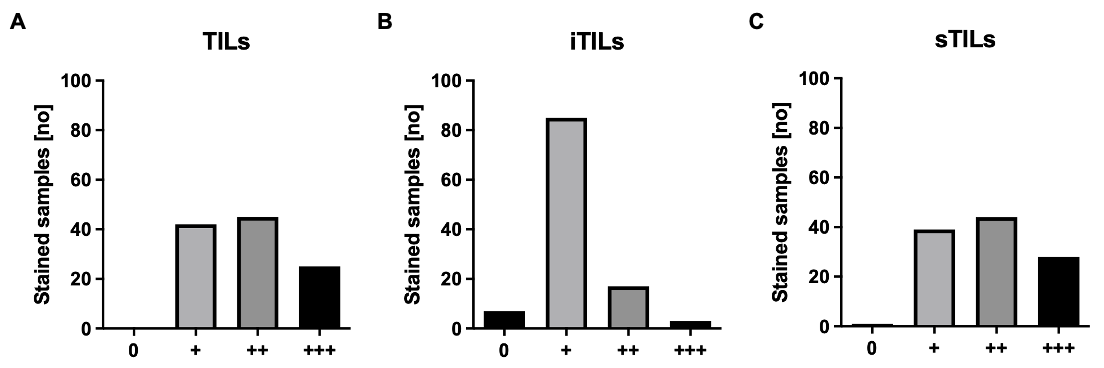


**Supplementary Figure 1**: Semi-quantitative scoring of tumour-infiltrating lymphocytes (A) and differentiation into intratumoural TILs (B) and stromal TILs (C) based on H&E slides.
