# Supplementary material for: Characterisation of tumour-immune phenotypes and PD-L1 positivity in squamous bladder cancer
Source: BMC Cancer. 2023 Feb 1;23:113. doi: 10.1186/s12885-023-10576-0 (PMC9890720; doi:10.1186/s12885-023-10576-0)
Supplement: Supplementary file 2 — Additional file 2: Supplementary Figure 2. Quantification of stained immune cells in stromal and tumour areas of three tumour cores by n=9 independent individuals. [file 12885_2023_10576_MOESM2_ESM.docx]

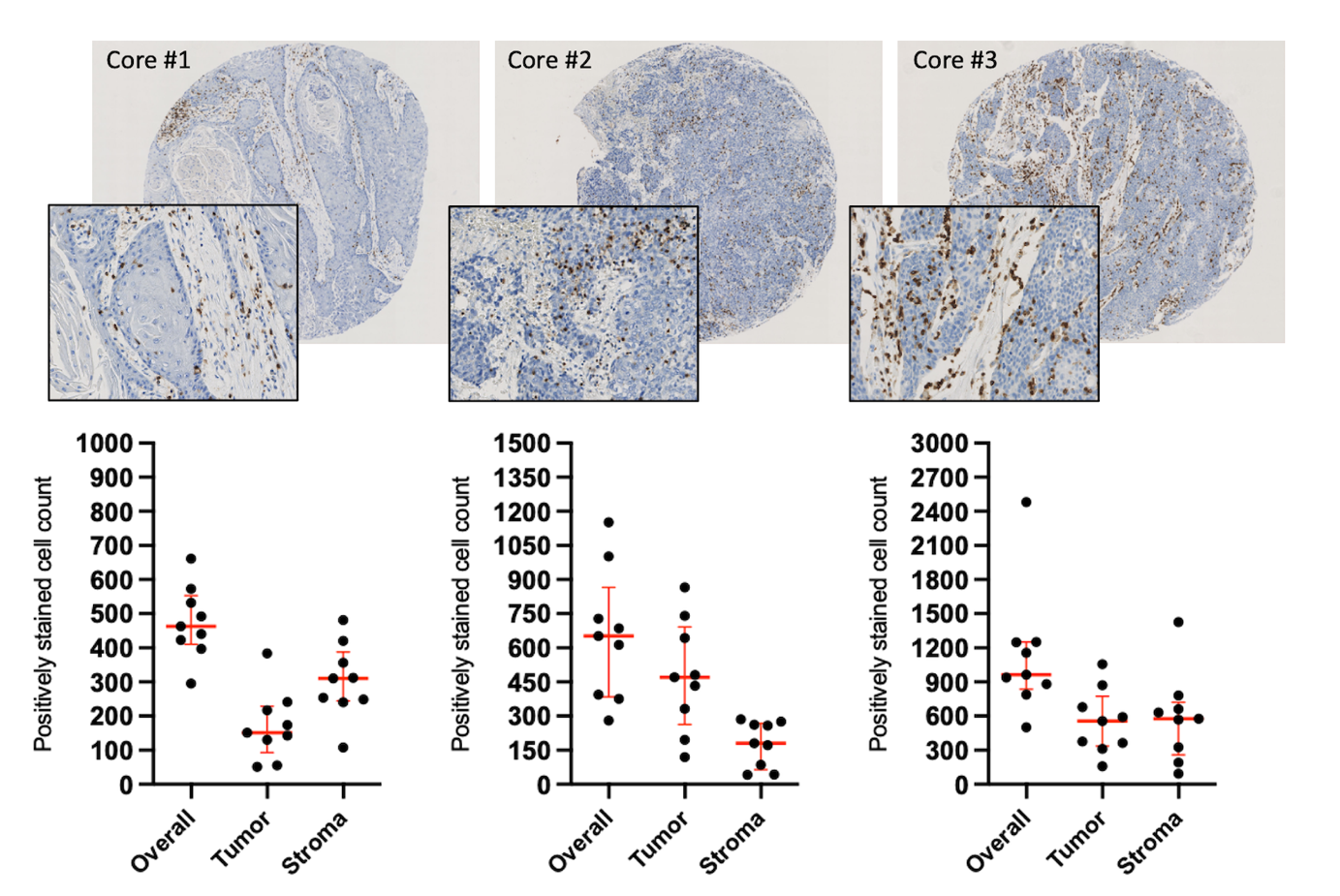


**Supplementary Figure 2:** Quantification of stained immune cells in stromal and tumour areas of three tumour cores by n=9 independent individuals.
