# Supplementary material for: Characterisation of tumour-immune phenotypes and PD-L1 positivity in squamous bladder cancer
Source: BMC Cancer. 2023 Feb 1;23:113. doi: 10.1186/s12885-023-10576-0 (PMC9890720; doi:10.1186/s12885-023-10576-0)
Supplement: Supplementary file 3 — Additional file 3: Supplementary Figure 3. Immunohistochemical staining of Ki67 and Perforin. Positively stained cell density quantified by QuPath in tumour and stroma for Ki67 and iTILs and sTILs for Perforin. [file 12885_2023_10576_MOESM3_ESM.docx]

**
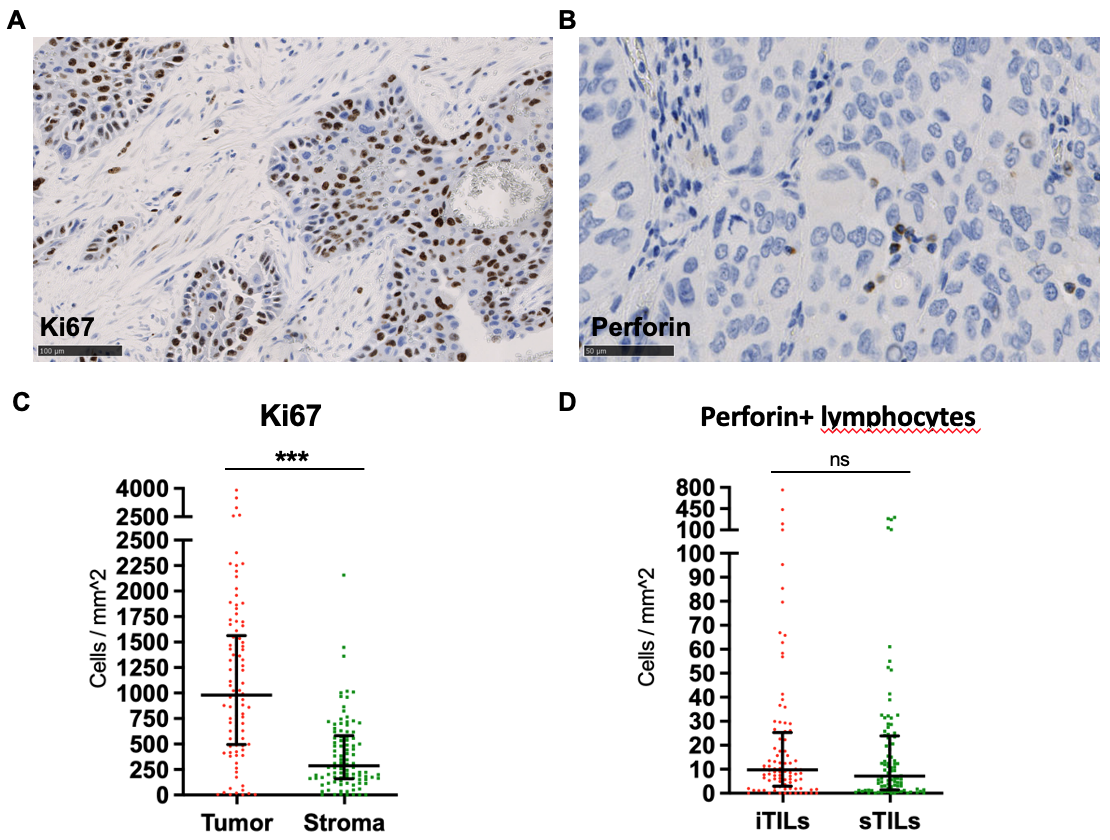
**

**Supplementary Figure 3**: Immunohistochemical staining of Ki67 (A) and Perforin (B). Black scale bar: 100 µm (Ki67); 50 µm (Perforin). Positively stained cell density quantified by QuPath in tumour and stroma for Ki67 (C) and iTILs and sTILs for Perforin (D).
