# Supplementary material for: Characterisation of tumour-immune phenotypes and PD-L1 positivity in squamous bladder cancer
Source: BMC Cancer. 2023 Feb 1;23:113. doi: 10.1186/s12885-023-10576-0 (PMC9890720; doi:10.1186/s12885-023-10576-0)
Supplement: Supplementary file 4 — Additional file 4: Supplementary Table 1. Study characteristics of the tissue microarray cohort. Supplementary Table 2. Antibodies used for immunohistochemistry. Supplementary Table 3. QuPath cell detection parameters. Supplementary Table 4. Semi-quantitative scoring of TILs in pure and mix SCC. Supplementary Table 5. Clinico-pathological parameters associated with Perforin density. Supplementary Table 6. Clinico-pathological parameters associated with Ki67 density. Supplementary Table 7. Clinico-pathological parameters associated with CD3 density. Supplementary Table 8. Clinico-pathological parameters associated with CD4 density. Supplementary Table 9. Clinico-pathological parameters associated with CD68 density. Supplementary Table 10. Clinico-pathological parameters associated with CD79A density. Supplementary Table 11. Clinico-pathological parameters associated with CD163 density. Supplementary Table 12. Clinico-pathological parameters associated with neutrophil granulocytes density. Supplementary Table 13. PD-L1 22C3 CPS and FGFR3 mutation status associated with Ki67 density. Supplementary Table 14. PD-L1 22C3 tumour cells expression associated with tumour-immune phenotypes. Supplementary Table 15. Nectin-4, Trop-2 expression and PIK3CA mutation status association with immune cell densities. [file 12885_2023_10576_MOESM4_ESM.docx]

**Supplementary Tables**

**Supplementary Table 1.** Study characteristics of the tissue microarray cohort

|  | **Bladder SCC n (%)** | |
| --- | --- | --- |
|  | **Pure SCC** | **Mix SCC** |
| **Total number of cases** | **68** | **46** |
| **Gender** |  |  |
| Male | 32 (49.2) | 19 (43.2) |
| Female | 33 (50.8) | 25 (56.8) |
| **Not available** | 3 | 2 |
| **Age** |  |  |
| Minimum - Maximum | 33-91 | 34-88 |
| Median  **Not available**  **Stage**  pT1  pT2  pT3  pT4  **Not available** | 64  2  1 (1.6)  10 (16.4)  37 (60.7)  13 (21.3)  7 | 71  2  0 (0)  4 (9.1)  35 (79.5)  5 (11.4)  2 |
| **Grading WHO 1973** |  |  |
| G1 | 1 (1.6) | 0 (0) |
| G2  G3  G4  **Not available** | 24 (37.5)  39 (60.9)  0 (0)  4 | 7 (15.9)  35 (79.5)  2 (4.5)  2 |
| **Nodal status** |  |  |
| pN0 | 39 (58.2) | 27 (58.7) |
| pN+ | 11 (16.4) | 11 (23.9) |
| pNx  **Not available** | 17 (25.4)  1 | 8 (17.4)  0 |
| **Lymphatic invasion** |  |  |
| L0 | 23 (34.3) | 16 (34.8) |
| L1 | 10 (14.9) | 13 (28.3) |
| Lx  **Not available** | 34 (50.7)  1 | 17 (37.0)  0 |

| **Supplementary Table 2.** Antibodies used for immunohistochemistry | | | |  |
| --- | --- | --- | --- | --- |
| **Antibody** | **Company** | **Clone** | **Dilution** | **pH conditions** |
| CD3 | Dako | UCHT1 | Ready to use | 6.1 |
| CD4 | Dako | 4B12 | Ready to use | 9.0 |
| CD8 | Dako | C8/144B | Ready to use | 6.1 |
| CD56 | Dako | 123C3 | Ready to use | 6.1 |
| CD68 | Dako | PG-M1 | Ready to use | 6.1 |
| CD79A | Dako | JCB117 | Ready to use | 9.0 |
| CD163 | Cell Marque | MRQ-26 | 01:20 | 9.0 |
| Ki67 | Agilent | MIB-1 | Ready to use | 6.1 |
| Perforin | Thermo Scientific | 5B10 | 1:100 | 9.0 |

**Supplementary Table 3**: QuPath cell detection parameters

| **Parameter** | **CD3** | **CD4** | **CD8** | **CD56** | **CD68** | **CD79A** | **CD163** | **Chloro.** | **Ki67** | **Perforin** |
| --- | --- | --- | --- | --- | --- | --- | --- | --- | --- | --- |
| **Stain vectors** | Pick manually | Pick manually | Pick manually | Pick manually | Pick manually | Pick manually | Pick manually | Pick manually | Pick manually | Pick manually |
| **Detection image** | Hematoxylin OD | Hematoxylin OD | Hematoxylin OD | Hematoxylin OD | Hematoxylin OD | Hematoxylin OD | Hematoxylin OD | Hematoxylin OD | Hematoxylin OD | Hematoxylin OD |
| **Requested pixel size** | 0.5 µm | 0.5 µm | 0.5 µm | 0.5 µm | 0.5 µm | 0.5 µm | 0.5 µm | 0.5 µm | 0.5 µm | 0.5 µm |
| **Background radius** | 8 µm | 8 µm | 8 µm | 8 µm | 8 µm | 8 µm | 8 µm | 8 µm | 8 µm | 8 µm |
| **Median filter radius** | 0 µm | 0 µm | 0 µm | 0 µm | 0 µm | 0 µm | 0 µm | 0 µm | 0 µm | 0 µm |
| **Sigma** | 1.5 µm | 1.5 µm | 1.5 µm | 1.5 µm | 2 µm | 1.5 µm | 2 µm | 1.5 µm | 2 µm | 1.5 µm |
| **Minimum area** | 10 µm^2 | 10 µm^2 | 10 µm^2 | 10 µm^2 | 25 µm^2 | 10 µm^2 | 25 µm^2 | 10 µm^2 | 10 µm^2 | 10 µm^2 |
| **Maximum area** | 100 µm^2 | 100 µm^2 | 100 µm^2 | 100 µm^2 | 250 µm^2 | 100 µm^2 | 250 µm^2 | 100 µm^2 | 400 µm^2 | 100 µm^2 |
| **Intensity threshold** | 0.08 | 0.06 | 0.08 | 0.08 | 0.06 | 0.08 | 0.08 | 0.25 | 0.01 | 0.08 |
| **Max. background intensity** | 2 | 2 | 2 | 2 | 2 | 2 | 2 | 2 | 2 | 2 |
| **Split by shape** | On | On | On | On | On | On | On | On | On | On |
| **Exclude DAB** | Off | Off | Off | Off | Off | Off | Off | Off | Off | Off |
| **Cell expansion** | 1.5 µm | 1.5 µm | 1.5 µm | 1.5 µm | 3 µm | 1.5 µm | 3 µm | 1.5 µm | 3 µm | 1.5 µm |
| **Include cell nucleus** | On | On | On | On | On | On | On | On | On | On |
| **Smooth boundaries** | On | On | On | On | On | On | On | On | On | On |
| **Make measurements** | On | On | On | On | On | On | On | On | On | On |
| **Score compartment** | Nucleus DAB OD mean | Nucleus DAB OD mean | Nucleus DAB OD mean | Nucleus DAB OD mean | Nucleus DAB OD mean | Nucleus DAB OD mean | Nucleus DAB OD mean | Nucleus DAB OD mean | Nucleus DAB OD mean | Cytoplasm DAB OD mean |
| **Threshold 1+** | 0.2 | 0.14 | 0.14 | 0.18 | 0.18 | 0.18 | 0.2 | 0.3 | 0.2 | 0.12 |
| **Single Threshold** | On | On | On | On | On | On | On | On | On | On |

| **Supplementary Table 4:** | | | | | | | | | | | | | | | | | |  | |
| --- | --- | --- | --- | --- | --- | --- | --- | --- | --- | --- | --- | --- | --- | --- | --- | --- | --- | --- | --- |
| **Semi-quantitative scoring of TILs in pure and mix SCC** | | | | | | | | | | | | | | | | | |  |  |
|  |  |  |  |  |  |  |  |  |  |  |  |  |  |  |  |  |  |  |  |
|  | | | | **Tumour subtype** | | | | | | | | | | | | | |  |  |
|  | | | | ***n****^a^* | | | | **Pure** | | | | **Mix** | | | **P-value** | | |  |  |
| TILs | | | |  | | | |  | | | |  | | |  | | |  |  |
|  | | | 0 | 0 | | | | 0 | | | | 0 | | | 0.465^b^ | | |  |  |
|  | | | + | 42 | | | | 28 | | | | 14 | | |  |  |  |  |  |
|  | | | ++ | 45 | | | | 26 | | | | 19 | | |  |  |  |  |  |
|  | | | +++ | 25 | | | | 13 | | | | 12 | | |  |  |  |  |  |
| iTILs | | | | | | | | | | | | | | |  | | |  |  |
|  | | | 0 | 7 | | | | 5 | | | | 2 | | | 0.878^c^ | | |  |  |
|  | | | + | 85 | | | | 51 | | | | 34 | | |  |  |  |  |  |
|  | | | ++ | 17 | | | | 9 | | | | 8 | | |  |  |  |  |  |
|  | | | +++ | 3 | | | | 2 | | | | 1 | | |  |  |  |  |  |
| sTILs | | | | | | | | | | | | | | |  | | |  |  |
|  | | | 0 | 1 | | | | 1 | | | | 0 | | | 0.827^c^ | | |  |  |
|  | | | + | 39 | | | | 25 | | | | 14 | | |  |  |  |  |  |
|  | | | ++ | 44 | | | | 26 | | | | 18 | | |  |  |  |  |  |
|  | | | +++ | 28 | | | | 15 | | | | 13 | | |  |  |  |  |  |
| ^a^Only patients with primary sq-BLCA were included; ^b^Pearson's chi-square test; ^c^Fisher’s exact test; Significant P-values are marked in bold face. Please note: sample numbers may vary between analyses due to limitations of usable TMA cores depending on the staining. | | | | | | | | | | | | | | | | | |  |  |
|  |  |  |  |  |  |  |  |  |  |  |  |  |  |  |  |  |  |  |  |
|  |  |  |  |  |  |  |  |  |  |  |  |  |  |  |  |  |  |  |  |
| **Supplementary Table 5:** | | | | | | | | | | | | | |  | | | |  |  |
| **Clinico-pathological parameters associated with Perforin density** | | | | | | | | | | | | | | | | | |  |  |
|  |  |  |  |  |  |  |  |  |  |  |  |  |  |  |  |  |  |  |  |
|  | | | | | | **Perforin density**^b^ | | | | | | | | | | | |  |  |
|  | | | | | | ***n****^a^* | | **low** | | | **high** | | | **P-value**^c^ | | | |  |  |
| ***Parameter:*** | | | | | | | | | | | | | |  | | | |  |  |
| Age at diagnosis | | | | | |  | |  | | |  | | |  | | | |  |  |
|  | <68 years | | | | | 45 | | 23 | | | 22 | | | 0.917 | | | |  |  |
|  | ≥68 years | | | | | 44 | | 22 | | | 22 | | |  |  |  |  |  |  |
| Gender | | | | | | | | | | | | | |  | | | |  |  |
|  | male | | | | | 42 | | 22 | | | 20 | | | 0.746 | | | |  |  |
|  | female | | | | | 47 | | 23 | | | 24 | | |  |  |  |  |  |  |
| Tumour subtype | | | | | | | | | | | | | |  | | | |  |  |
|  | pure SCC | | | | | 53 | | 33 | | | 20 | | | **0.006** | | | |  |  |
|  | mix SCC | | | | | 39 | | 13 | | | 26 | | |  |  |  |  |  |  |
| Histological tumour grade | | | | | |  | |  | | |  | | |  | | | |  |  |
|  | G1-G2 | | | | | 25 | | 13 | | | 12 | | | 0.760 | | | |  |  |
|  | G3-G4 | | | | | 62 | | 30 | | | 32 | | |  |  |  |  |  |  |
| Tumour stage | | | | | |  | |  | | |  | | |  | | | |  |  |
|  | pT1-pT2 | | | | | 11 | | 3 | | | 8 | | | 0.136 | | | |  |  |
|  | pT3-pT4 | | | | | 74 | | 38 | | | 36 | | |  |  |  |  |  |  |
| Nodal status | | | | | |  | |  | | |  | | |  | | | |  |  |
|  | pN0 | | | | | 54 | | 24 | | | 30 | | | 0.419 | | | |  |  |
|  | pN+ | | | | | 20 | | 11 | | | 9 | | |  |  |  |  |  |  |
| Lymphatic invasion | | | | | |  | |  | | |  | | |  | | | |  |  |
|  | L0 | | | | | 33 | | 16 | | | 17 | | | 0.357 | | | |  |  |
|  | L1 | | | | | 16 | | 10 | | | 6 | | |  |  |  |  |  |  |
| ^a^Only patients with primary sq-BLCA were included; ^b^density in cells / mm^2^; median value of the overall sample (9.0 / mm^2^) as cut-off for low and high density; ^c^Pearson's chi-square test; Significant P-values are marked in bold face. Please note: sample numbers may vary between analyses due to limitations of usable TMA cores depending on the staining. | | | | | | | | | | | | | | | | | |  |  |
|  |  |  |  |  |  |  |  |  |  |  |  |  |  |  |  |  |  |  |  |
|  |  |  |  |  |  |  |  |  |  |  |  |  |  |  |  |  |  |  |  |
| **Supplementary Table 6:** | | | | | | | | | | | | |  | | | |  |  |  |
| **Clinico-pathological parameters associated with Ki67 density** | | | | | | | | | | | | | | | | |  |  |  |
|  |  |  |  |  |  |  |  |  |  |  |  |  |  |  |  |  |  |  |  |
|  | | | | | **Ki67 density**^b^ | | | | | | | | | | | |  |  |  |
|  | | | | | ***n****^a^* | | **low** | | | **high** | | | **P-value**^c^ | | | |  |  |  |
| ***Parameter:*** | | | | | | | | | | | | |  | | | |  |  |  |
| Age at diagnosis | | | | |  | |  | | |  | | |  | | | |  |  |  |
|  | <68 years | | | | 44 | | 19 | | | 25 | | | 0.295 | | | |  |  |  |
|  | ≥68 years | | | | 50 | | 27 | | | 23 | | |  |  |  |  |  |  |  |
| Gender | | | | | | | | | | | | |  | | | |  |  |  |
|  | male | | | | 43 | | 25 | | | 18 | | | 0.101 | | | |  |  |  |
|  | female | | | | 51 | | 21 | | | 30 | | |  |  |  |  |  |  |  |
| Tumour subtype | | | | | | | | | | | | |  | | | |  |  |  |
|  | pure SCC | | | | 58 | | 31 | | | 27 | | | 0.411 | | | |  |  |  |
|  | mix SCC | | | | 40 | | 18 | | | 22 | | |  |  |  |  |  |  |  |
| Histological tumour grade | | | | |  | |  | | |  | | |  | | | |  |  |  |
|  | G1-G2 | | | | 28 | | 19 | | | 9 | | | **0.016** | | | |  |  |  |
|  | G3-G4 | | | | 64 | | 26 | | | 38 | | |  |  |  |  |  |  |  |
| Tumour stage | | | | |  | |  | | |  | | |  | | | |  |  |  |
|  | pT1-pT2 | | | | 13 | | 4 | | | 9 | | | 0.171 | | | |  |  |  |
|  | pT3-pT4 | | | | 76 | | 39 | | | 37 | | |  |  |  |  |  |  |  |
| Nodal status | | | | |  | |  | | |  | | |  | | | |  |  |  |
|  | pN0 | | | | 55 | | 25 | | | 30 | | | 0.464 | | | |  |  |  |
|  | pN+ | | | | 20 | | 11 | | | 9 | | |  |  |  |  |  |  |  |
| Lymphatic invasion | | | | |  | |  | | |  | | |  | | | |  |  |  |
|  | L0 | | | | 32 | | 11 | | | 21 | | | **0.019** | | | |  |  |  |
|  | L1 | | | | 19 | | 13 | | | 6 | | |  |  |  |  |  |  |  |
| ^a^Only patients with primary sq-BLCA were included; ^b^density in cells / mm^2^; median value of the overall sample (509.5 / mm^2^) as cut-off for low and high density; ^c^Pearson's chi-square test; Significant P-values are marked in bold face. Please note: sample numbers may vary between analyses due to limitations of usable TMA cores depending on the staining. | | | | | | | | | | | | | | | | |  |  |  |
|  |  |  |  |  |  |  |  |  |  |  |  |  |  |  |  |  |  |  |  |
| **Supplementary Table 7:** | | | | | | | | | | | | | | | | | |  | |
| **Clinico-pathological parameters associated with CD3 density** | | | | | | | | | | | | | | | | | |  |  |
|  |  |  |  |  |  |  |  |  |  |  |  |  |  |  |  |  |  |  |  |
|  | | | | | | **CD3 density**^b^ | | | | | | | | | | | |  |  |
|  | | | | | | ***n****^a^* | | | **low** | | **high** | | | | | **P-value**^c^ | |  |  |
| ***Parameter:*** | | | | | | | | | | | | | | | |  | |  |  |
| Age at diagnosis | | | | | |  | | |  | |  | | | | |  | |  |  |
|  | | <68 years | | | | 54 | | | 23 | | 31 | | | | | 0.178 | |  |  |
|  | | ≥68 years | | | | 54 | | | 30 | | 24 | | | | |  |  |  |  |
| Gender | | | | | | | | | | | | | | | |  | |  |  |
|  | | male | | | | 51 | | | 29 | | 22 | | | | | 0.148 | |  |  |
|  | | female | | | | 56 | | | 24 | | 32 | | | | |  |  |  |  |
| Tumour subtype | | | | | | | | | | | | | | | |  | |  |  |
|  | | pure SCC | | | | 67 | | | 37 | | 30 | | | | | 0.215 | |  |  |
|  | | mix SCC | | | | 44 | | | 19 | | 25 | | | | |  |  |  |  |
| Histological tumour grade | | | | | | | | | | | | | | | |  | |  |  |
|  | | G1-G2 | | | | 32 | | | 17 | | 15 | | | | | 0.497 | |  |  |
|  | | G3-G4 | | | | 74 | | | 34 | | 40 | | | | |  |  |  |  |
| Tumour stage | | | | | | | | | | | | | | | |  | |  |  |
|  | | pT1-pT2 | | | | 15 | | | 7 | | 8 | | | | | 0.996 | |  |  |
|  | | pT3-pT4 | | | | 88 | | | 41 | | 47 | | | | |  |  |  |  |
| Nodal status | | | | | | | | | | | | | | | |  | |  |  |
|  | | pN0 | | | | 65 | | | 31 | | 34 | | | | | 0.442 | |  |  |
|  | | pN+ | | | | 21 | | | 8 | | 13 | | | | |  |  |  |  |
| Lymphatic invasion | | | | | | | | | | | | | | | |  | |  |  |
|  | | L0 | | | | 39 | | | 16 | | 23 | | | | | 0.737 | |  |  |
|  | | L1 | | | | 22 | | | 10 | | 12 | | | | |  |  |  |  |
| ^a^Only patients with primary sq-BLCA were included; ^b^density in cells / mm^2^; median value of the overall sample (582.3 / mm^2^) as cut-off for low and high density; ^c^Pearson's chi-square test; Significant P-values are marked in bold face. Please note: sample numbers may vary between analyses due to limitations of usable TMA cores depending on the staining. | | | | | | | | | | | | | | | | | |  |  |
|  |  |  |  |  |  |  |  |  |  |  |  |  |  |  |  |  |  |  |  |
|  |  |  |  |  |  |  |  |  |  |  |  |  |  |  |  |  |  |  |  |
| \| **Supplementary Table 8:** \| \| \| \| \|  \| \| --- \| --- \| --- \| --- \| --- \| --- \| \| **Clinico-pathological parameters associated with CD4 density** \| \| \| \| \| \| \|  \| \|  \| \| **CD4 density**^b^ \| \| \| \|  \| \|  \| \| ***n****^a^* \| **low** \| **high** \| **P-value**^c^ \|  \| \| ***Parameter:*** \| \| \| \| \|  \|  \| \| Age at diagnosis \| \|  \|  \|  \|  \|  \| \|  \| <68 years \| 50 \| 24 \| 26 \| 0.915 \|  \| \|  \| ≥68 years \| 53 \| 26 \| 27 \|  \| \| Gender \| \| \| \| \|  \|  \| \|  \| male \| 49 \| 28 \| 21 \| 0.115 \|  \| \|  \| female \| 53 \| 22 \| 31 \|  \| \| Tumour subtype \| \| \| \| \|  \|  \| \|  \| pure SCC \| 66 \| 36 \| 30 \| 0.229 \|  \| \|  \| mix SCC \| 40 \| 17 \| 23 \|  \| \| Histological tumour grade \| \|  \|  \|  \|  \|  \| \|  \| G1-G2 \| 31 \| 18 \| 13 \| 0.201 \|  \| \|  \| G3-G4 \| 70 \| 31 \| 39 \|  \| \| Tumour stage \| \|  \|  \|  \|  \|  \| \|  \| pT1-pT2 \| 15 \| 10 \| 5 \| 0.096 \|  \| \|  \| pT3-pT4 \| 83 \| 36 \| 47 \|  \| \| Nodal status \| \|  \|  \|  \|  \|  \| \|  \| pN0 \| 61 \| 30 \| 31 \| 0.617 \|  \| \|  \| pN+ \| 21 \| 9 \| 12 \|  \| \| Lymphatic invasion \| \|  \|  \|  \|  \|  \| \|  \| L0 \| 37 \| 14 \| 23 \| 0.873 \|  \| \|  \| L1 \| 20 \| 8 \| 12 \|  \| \| ^a^Only patients with primary sq-BLCA were included; ^b^density in cells / mm^2^; median value of the overall sample (141.5 / mm^2^) as cut-off for low and high density; ^c^Pearson's chi-square test; Significant P-values are marked in bold face. Please note: sample numbers may vary between analyses due to limitations of usable TMA cores depending on the staining. \| \| \| \| \| \|  \| \|  \| \|  \|  \| **Supplementary Table 9:** \| \| \| \| \|  \| \| --- \| --- \| --- \| --- \| --- \| --- \| \| **Clinico-pathological parameters associated with CD68 density** \| \| \| \| \| \| \|  \| \|  \| \| **CD68 density**^b^ \| \| \| \|  \| \|  \| \| ***n****^a^* \| **low** \| **high** \| **P-value**^c^ \|  \| \| ***Parameter:*** \| \| \| \| \|  \|  \| \| Age at diagnosis \| \|  \|  \|  \|  \|  \| \|  \| <68 years \| 52 \| 25 \| 27 \| 0.843 \|  \| \|  \| ≥68 years \| 54 \| 27 \| 27 \|  \| \| Gender \| \| \| \| \|  \|  \| \|  \| male \| 49 \| 23 \| 26 \| 0.620 \|  \| \|  \| female \| 56 \| 29 \| 27 \|  \| \| Tumour subtype \| \| \| \| \|  \|  \| \|  \| pure SCC \| 66 \| 33 \| 33 \| 0.906 \|  \| \|  \| mix SCC \| 43 \| 22 \| 21 \|  \| \| Histological tumour grade \| \|  \|  \|  \|  \|  \| \|  \| G1-G2 \| 31 \| 16 \| 15 \| 0.638 \|  \| \|  \| G3-G4 \| 73 \| 34 \| 39 \|  \| \| Tumour stage \| \|  \|  \|  \|  \|  \| \|  \| pT1-pT2 \| 15 \| 7 \| 8 \| 0.991 \|  \| \|  \| pT3-pT4 \| 86 \| 40 \| 46 \|  \| \| Nodal status \| \|  \|  \|  \|  \|  \| \|  \| pN0 \| 64 \| 30 \| 34 \| 0.953 \|  \| \|  \| pN+ \| 21 \| 10 \| 11 \|  \| \| Lymphatic invasion \| \|  \|  \|  \|  \|  \| \|  \| L0 \| 38 \| 17 \| 21 \| 0.474 \|  \| \|  \| L1 \| 20 \| 7 \| 13 \|  \| \| ^a^Only patients with primary sq-BLCA were included; ^b^density in cells / mm^2^; median value of the overall sample (493.0 / mm^2^) as cut-off for low and high density; ^c^Pearson's chi-square test; Significant P-values are marked in bold face. Please note: sample numbers may vary between analyses due to limitations of usable TMA cores depending on the staining. \| \| \| \| \| \|  \| \|  \| \|  \|  \| **Supplementary Table 10:** \| \| \| \| \|  \| \| --- \| --- \| --- \| --- \| --- \| --- \| \| **Clinico-pathological parameters associated with CD79A density** \| \| \| \| \| \| \|  \| \|  \| \| **CD79A density**^b^ \| \| \| \|  \| \|  \| \| ***n****^a^* \| **low** \| **high** \| **P-value**^c^ \|  \| \| ***Parameter:*** \| \| \| \| \|  \|  \| \| Age at diagnosis \| \|  \|  \|  \|  \|  \| \|  \| <68 years \| 54 \| 21 \| 33 \| **0.034** \|  \| \|  \| ≥68 years \| 54 \| 32 \| 22 \|  \| \| Gender \| \| \| \| \|  \|  \| \|  \| male \| 51 \| 28 \| 23 \| 0.289 \|  \| \|  \| female \| 56 \| 25 \| 31 \|  \| \| Tumour subtype \| \| \| \| \|  \|  \| \|  \| pure SCC \| 67 \| 35 \| 32 \| 0.642 \|  \| \|  \| mix SCC \| 44 \| 21 \| 23 \|  \| \| Histological tumour grade \| \|  \|  \|  \|  \|  \| \|  \| G1-G2 \| 32 \| 13 \| 19 \| 0.253 \|  \| \|  \| G3-G4 \| 74 \| 39 \| 35 \|  \| \| Tumour stage \| \|  \|  \|  \|  \|  \| \|  \| pT1-pT2 \| 15 \| 6 \| 9 \| 0.525 \|  \| \|  \| pT3-pT4 \| 88 \| 43 \| 45 \|  \| \| Nodal status \| \|  \|  \|  \|  \|  \| \|  \| pN0 \| 65 \| 33 \| 32 \| 0.164 \|  \| \|  \| pN+ \| 21 \| 7 \| 14 \|  \| \| Lymphatic invasion \| \|  \|  \|  \|  \|  \| \|  \| L0 \| 39 \| 16 \| 23 \| 0.175 \|  \| \|  \| L1 \| 22 \| 13 \| 9 \|  \| \| ^a^Only patients with primary sq-BLCA were included; ^b^density in cells / mm^2^; median value of the overall sample (85.9 / mm^2^) as cut-off for low and high density; ^c^Pearson's chi-square test; Significant P-values are marked in bold face. Please note: sample numbers may vary between analyses due to limitations of usable TMA cores depending on the staining. \| \| \| \| \| \|  \| \|  \| \|  \|  \| **Supplementary Table 11:** \| \| \| \| \|  \| \| --- \| --- \| --- \| --- \| --- \| --- \| \| **Clinico-pathological parameters associated with CD163 density** \| \| \| \| \| \| \|  \| \|  \| \| **CD163 density**^b^ \| \| \| \|  \| \|  \| \| ***n****^a^* \| **low** \| **high** \| **P-value**^c^ \|  \| \| ***Parameter:*** \| \| \| \| \|  \|  \| \| Age at diagnosis \| \|  \|  \|  \|  \|  \| \|  \| <68 years \| 52 \| 29 \| 23 \| 0.244 \|  \| \|  \| ≥68 years \| 54 \| 24 \| 30 \|  \| \| Gender \| \| \| \| \|  \|  \| \|  \| male \| 50 \| 24 \| 26 \| 0.628 \|  \| \|  \| female \| 55 \| 29 \| 26 \|  \| \| Tumour subtype \| \| \| \| \|  \|  \| \|  \| pure SCC \| 66 \| 38 \| 28 \| 0.066 \|  \| \|  \| mix SCC \| 43 \| 17 \| 26 \|  \| \| Histological tumour grade \| \|  \|  \|  \|  \|  \| \|  \| G1-G2 \| 32 \| 18 \| 14 \| 0.327 \|  \| \|  \| G3-G4 \| 72 \| 33 \| 39 \|  \| \| Tumour stage \| \|  \|  \|  \|  \|  \| \|  \| pT1-pT2 \| 15 \| 5 \| 10 \| 0.202 \|  \| \|  \| pT3-pT4 \| 86 \| 44 \| 42 \|  \| \| Nodal status \| \|  \|  \|  \|  \|  \| \|  \| pN0 \| 63 \| 32 \| 31 \| 0.529 \|  \| \|  \| pN+ \| 21 \| 9 \| 12 \|  \| \| Lymphatic invasion \| \|  \|  \|  \|  \|  \| \|  \| L0 \| 37 \| 17 \| 20 \| 0.706 \|  \| \|  \| L1 \| 22 \| 9 \| 13 \|  \| \| ^a^Only patients with primary sq-BLCA were included; ^b^density in cells / mm^2^; median value of the overall sample (462.1 / mm^2^) as cut-off for low and high density; ^c^Pearson's chi-square test; Significant P-values are marked in bold face. Please note: sample numbers may vary between analyses due to limitations of usable TMA cores depending on the staining. \| \| \| \| \| \|  \| \|  \| \|  \|  \| **Supplementary Table 12:** \| \| \| \| \|  \| \| --- \| --- \| --- \| --- \| --- \| --- \| \| **Clinico-pathological parameters associated with neutrophil granulocytes density** \| \| \| \| \| \| \|  \| \|  \| \| **Neutrophil granulocytes density**^b^ \| \| \| \|  \| \|  \| \| ***n****^a^* \| **low** \| **high** \| **P-value**^c^ \|  \| \| ***Parameter:*** \| \| \| \| \|  \|  \| \| Age at diagnosis \| \|  \|  \|  \|  \|  \| \|  \| <68 years \| 47 \| 22 \| 25 \| 0.680 \|  \| \|  \| ≥68 years \| 51 \| 26 \| 25 \|  \| \| Gender \| \| \| \| \|  \|  \| \|  \| male \| 47 \| 20 \| 27 \| 0.222 \|  \| \|  \| female \| 51 \| 28 \| 23 \|  \| \| Tumour subtype \| \| \| \| \|  \|  \| \|  \| pure SCC \| 61 \| 29 \| 32 \| 0.545 \|  \| \|  \| mix SCC \| 41 \| 22 \| 19 \|  \| \| Histological tumour grade \| \|  \|  \|  \|  \|  \| \|  \| G1-G2 \| 28 \| 16 \| 12 \| 0.369 \|  \| \|  \| G3-G4 \| 68 \| 32 \| 36 \|  \| \| Tumour stage \| \|  \|  \|  \|  \|  \| \|  \| pT1-pT2 \| 13 \| 7 \| 6 \| 0.797 \|  \| \|  \| pT3-pT4 \| 80 \| 40 \| 40 \|  \| \| Nodal status \| \|  \|  \|  \|  \|  \| \|  \| pN0 \| 59 \| 28 \| 31 \| 0.844 \|  \| \|  \| pN+ \| 20 \| 10 \| 10 \|  \| \| Lymphatic invasion \| \|  \|  \|  \|  \|  \| \|  \| L0 \| 36 \| 18 \| 18 \| 0.853 \|  \| \|  \| L1 \| 19 \| 10 \| 9 \|  \| \| ^a^Only patients with primary sq-BLCA were included; ^b^density in cells / mm^2^; median value of the overall sample (163.6 / mm^2^) as cut-off for low and high density; ^c^Pearson's chi-square test; Significant P-values are marked in bold face. Please note: sample numbers may vary between analyses due to limitations of usable TMA cores depending on the staining. \| \| \| \| \| \|  \| \|  \| \|  \| | | | | | | | | | | | | | | | | | |  | |

| **Supplementary Table 13:** | | | | |  |
| --- | --- | --- | --- | --- | --- |
| **PD-L1 22C3 CPS and FGFR3 mutation status associated with Ki67 density** | | | | | |
|  |  |  |  |  |  |
|  | | **Ki67 density^b^** | | | |
|  | | ***n****^a^* | **low** | **high** | **P-value**^c^ |
| ***Markers:*** | | | | |  |
| PD-L1 CPS^d^ | |  |  |  |  |
|  | <10 | 77 | 42 | 35 | **0.049** |
|  | ≥10 | 10 | 2 | 8 |  |
| FGFR3 mutation | | | | |  |
|  | Negative | 45 | 20 | 25 | 1.000 |
|  | Positive | 7 | 3 | 4 |  |
| ^a^Only patients with primary sq-BLCA were included; ^b^density in cells / mm^2^; median value of the overall sample (509.5/mm^2^) as cut-off for low and high density; ^c^Fisher’s exact test; ^d^Combined positive score (CPS) according to Kulangara et al. (23); Significant P-values are marked in bold face. Please note: sample numbers may vary between analyses due to limitations of usable TMA cores depending on the staining. | | | | | |
|  |  |  |  |  |  |
|  |  |  |  |  |  |

| **Supplementary Table 14: PD-L1 22C3 tumour cells expression associated with tumour-immune phenotypes** | | | | | |
| --- | --- | --- | --- | --- | --- |
|  |  |  |  |  |  |
|  | | **PD-L1 22C3 expression (% tumour cells)^b^** | | | |
|  | | ***n****^a^* | **TPS≤0** | **TPS>0** | **P-value**^c^ |
| ***Immune cell markers:*** | | | | |  |
|  |  |  |  |  |  |
| Immune topography T-cells (CD3/CD4) | |  |  |  |  |
|  |  |  |  |  |  |
|  | hot | 28 | 21 | 7 | 0.053 |
|  | cold | 27 | 26 | 1 |  |
|  | excluded | 38 | 34 | 4 |  |
|  |  |  |  |  |  |
| Immune topography T-cells (CD3/CD8) | |  |  |  |  |
|  |  |  |  |  |  |
|  | hot | 29 | 22 | 7 | 0.098 |
|  | cold | 29 | 27 | 2 |  |
|  | excluded | 34 | 31 | 3 |  |
|  |  |  |  |  |  |
| Immune topography B- cells (CD79A) | |  |  |  |  |
|  |  |  |  |  |  |
|  | hot | 7 | 4 | 3 | **0.039** |
|  | cold | 9 | 8 | 1 |  |
|  | excluded | 81 | 73 | 8 |  |
|  |  |  |  |  |  |
| Immune topography macrophages (CD68/CD163) | |  |  |  |  |
|  |  |  |  |  |  |
|  | hot | 28 | 20 | 8 | **0.007** |
|  | cold | 30 | 27 | 3 |  |
|  | excluded | 37 | 36 | 1 |  |
|  |  |  |  |  |  |
| Immune topography all immune cells | |  |  |  |  |
|  |  |  |  |  |  |
|  | hot | 28 | 20 | 8 | **0.011** |
|  | cold | 31 | 28 | 3 |  |
|  | excluded | 32 | 31 | 1 |  |
|  |  |  |  |  |  |
| ^a^Only patients with primary sq-BLCA were included; ^b^PDL1 expression as % of tumour cells (TPS); ^c^Pearson’s chi-square test; Significant P-values are marked in bold face. Please note: sample numbers may vary between analyses due to limitations of usable TMA cores depending on the staining. | | | | | |
|  |  |  |  |  |  |
|  |  |  |  |  |  |

| **Supplementary Table 15:** | | | | | |  |  |  |  |  |  |  |  |
| --- | --- | --- | --- | --- | --- | --- | --- | --- | --- | --- | --- | --- | --- |
| **Nectin-4, Trop-2 expression and PIK3CA mutation status association with immune cell densities** | | | | | | | | | | | | | |
|  | | **Nectin-4^b^** | | | | **Trop-2^b^** | | | | **PIK3CA** | | | |
|  | | ***n****^a^* | **low** | **high** | **P-value**^b^ | **n^a^** | **low** | **high** | **P-value^b^** | **n^a^** | **neg** | **pos** | **P-value^b^** |
| ***Immune cell markers:*** | | | | |  |  |  |  |  |  |  |  |  |
| CD3 | |  |  |  |  |  |  |  |  |  |  |  |  |
|  | ≤582.3/mm^2^ | 42 | 26 | 16 | 0.570 | 41 | 12 | 29 | 0.432 | 26 | 23 | 3 | 0.186 |
|  | >582.3/mm^2^ | 36 | 20 | 16 |  | 40 | 15 | 25 |  | 36 | 27 | 9 |  |
| CD4 | | | | |  |  |  |  |  |  |  |  |  |
|  | ≤141.5/mm^2^ | 38 | 25 | 13 | 0.509 | 39 | 14 | 25 | 0.877 | 29 | 25 | 4 | 0.171 |
|  | >141.5/mm^2^ | 36 | 21 | 15 |  | 38 | 13 | 25 |  | 28 | 20 | 8 |  |
| CD8 | | | | |  |  |  |  |  |  |  |  |  |
|  | ≤291.6/mm^2^ | 38 | 25 | 13 | 0.448 | 39 | 12 | 27 | 0.516 | 25 | 22 | 3 | 0.217 |
|  | >291.6/mm^2^ | 35 | 20 | 15 |  | 37 | 14 | 23 |  | 32 | 24 | 8 |  |
| CD68 | |  |  |  |  |  |  |  |  |  |  |  |  |
|  | ≤493.0/mm^2^ | 44 | 29 | 15 | 0.392 | 43 | 14 | 29 | 0.672 | 28 | 23 | 5 | 0.698 |
|  | >493.0/mm^2^ | 32 | 18 | 14 |  | 35 | 13 | 22 |  | 32 | 25 | 7 |  |
| CD79A | |  |  |  |  |  |  |  |  |  |  |  |  |
|  | ≤85.9/mm^2^ | 42 | 24 | 18 | 0.722 | 43 | 15 | 28 | 0.753 | 30 | 25 | 5 | 0.604 |
|  | >85.9/mm^2^ | 36 | 22 | 14 |  | 38 | 12 | 26 |  | 32 | 25 | 7 |  |
| CD163 | |  |  |  |  |  |  |  |  |  |  |  |  |
|  | ≤462.1/mm^2^ | 44 | 28 | 16 | 0.421 | 43 | 16 | 27 | 0.481 | 32 | 28 | 4 | 0.121 |
|  | >462.1mm^2^ | 33 | 18 | 15 |  | 37 | 11 | 26 |  | 28 | 20 | 8 |  |
| Perforin | | | | |  |  |  |  |  |  |  |  |  |
|  | ≤9.0/mm^2^ | 40 | 26 | 14 | 0.885 | 41 | 12 | 29 | 0.575 | 17 | 14 | 3 | 0.765 |
|  | >9.0/mm^2^ | 30 | 19 | 11 |  | 31 | 11 | 20 |  | 33 | 26 | 7 |  |
|  |  |  |  |  |  |  |  |  |  |  |  |  |  |
| ^a^Only patients with primary sq-BLCA were included; ^b^Expression scores based on IHC and dichotomized (Nectin-4: 0-1 (low), 2-3 (high); Trop-2: 0-2 (low), 3 (high)); ^c^Pearson’s chi-square test; Significant P-values are marked in bold face. Please note: sample numbers may vary between analyses due to limitations of usable TMA cores depending on the staining. | | | | | | | | | | | | | |
